# Using crystallography, topology and graph set analysis for the description of the hydrogen bond network of triamterene: a rational approach to solid form selection

**DOI:** 10.1186/s13065-017-0293-1

**Published:** 2017-07-13

**Authors:** David S. Hughes, Amit Delori, Abida Rehman, William Jones

**Affiliations:** 1 Department of Chemistry, University of Cambridge, Lensfield Road, Cambridge, CB2 1EW UK; 2 Strathclyde Institute of Pharmacy and Biomedical Sciences (SIPBS), University of Strathclyde, 161 Cathedral Street, Glasgow, G4 0RE UK

**Keywords:** Triamterene, Crystallography, Topology, Graph set analysis, Solid form selection

## Abstract

This study has demonstrated the use of crystallography, topology and graph set analysis in the description and classification of the complex hydrogen bonded network of triamterene. The aim is to give a brief overview of the methodology used to discuss the crystal structure of triamterene with a view to extending the study to include the solvates, cocrystals and salts of this compound.

**Electronic supplementary material:**

The online version of this article (doi:10.1186/s13065-017-0293-1) contains supplementary material, which is available to authorized users.

**Graphical abstract:**
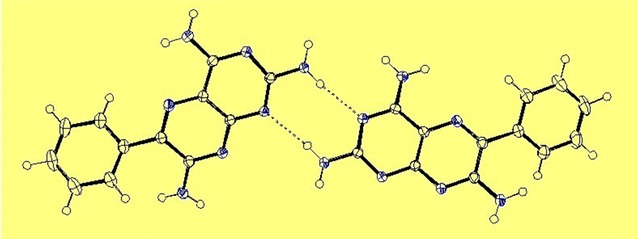
One of the structurally significant dimers (supramolecular synthons) of triamterene identified by this study

## Introduction

The Directed Assembly Network, an EPSRC Grand Challenge Network, was created in 2010 to build a wide-reaching community of scientists, engineers and industrial members that includes chemists, biologists, physicists, chemical engineers, mathematicians and computer scientists with a view to solving some of the most important technological (academic and industrial) challenges over the next 20–40 years through a structured programme of short, medium and long-term goals. A key document “Directed Assembly Network: Beyond the molecule—A Roadmap to Innovation” has been created by this community over several years of consultation and refinement. The latest version of this document published in 2016 outlines the programme and contains five main drivers (themes) for innovation [1]. The second theme involves controlling the nucleation and crystallization processes in the pharmaceutical and other fine chemical industries.

Briefly, the second theme aims to control the crystallization of active pharmaceutical ingredients (APIs) so that the therapeutic effect can be delivered safely and effectively to the target location in the body by the best possible route. At present, due to scientific and technological limitations the most active form is sometimes not manufactured due to compromises being made during the selection of the physical form. If the range of supramolecular structures for a given molecule could be known, along with a “wish-list” of optimum physical properties then this could revolutionise the drug discovery process. Knowledge of the complete range of solid forms available to a molecule and the ability to control the nucleation and crystallization of the best form using more economically favourable manufacturing processes should make it possible to obtain a “deliverable” product. For example, Delori et al. [2] recently used this knowledge to produce a range of (hydrogen peroxide and ammonia-free) hair products and so gain a strong foothold in the multi-billion dollar cosmetics industry.

This study aims to contribute to the second theme by focussing on the ability of triamterene, which is on the WHO list of the most important drugs in the clinic worldwide, to form potential solid forms through an in-depth understanding of its crystal structure. Previously, the molecules of triamterene have been described as being linked by an intricate and unusual network of hydrogen bonds [3] and this provides extra motivation for this study.

Central to the understanding of the creation of new forms is the ability to describe the differences and similarities found in a series of crystal structures. Sometimes helpful comparison of crystal structures is difficult since unit cells and space groups identified by crystallography are often defined by convention rather than to aid structural comparison. For hydrogen bonded structures the use of graph-set analysis has been suggested as a way of partially dealing with this problem [4]. As pointed out by Zolotarev et al. [5] (reference kindly provided by Reviewer) the prediction of synthons will have a significant impact on crystal structure and physical property prediction.

In this contribution, a combination of crystallography, hydrogen bond chemical connectivity, topology and graph-set analysis is used to describe and understand the crystal structure of triamterene with a view to implementing the method to alternative *analogue* and multicomponent solid forms. Of particular interest is the use of topology and graph-set notation for the enumeration and classification of hydrogen bonds in a complex system.

**Scheme 1:**
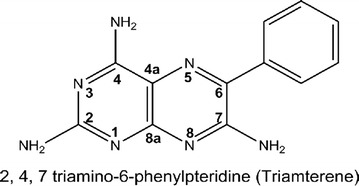
The triamterene molecule showing the IUPAC numbering scheme used for pteridine-like molecules

Triamterene (Scheme 1) is a valuable potassium sparing diuretic and a modest dihydrofolate reductase (DHFR) inhibitor. A current challenge in the pharmaceutical development of this drug is to improve its solubility without compromising stability and other valuable properties.

Available thermochemical and solubility data show that triamterene has a high melting point (327.31 °C) and is insoluble in water or methanol but sparingly soluble in 1-octanol, DMF or DMSO.

Calculated p*Ka* data show the ring nitrogen atom (N1) to be the most basic with a p*Ka* of 5.93 and the ring nitrogen atom (N5) with a p*Ka* of −2.49 to be the least basic site in this structure [6]. According to Etter [7, 8] not all combinations of donor and acceptor are equally likely, since strong hydrogen donors (strongly acidic hydrogens) will tend to form hydrogen bonds preferentially with strong hydrogen bond acceptors (atoms with available electron pairs). It is anticipated, therefore, that the nitrogen N1 of triamterene will participate preferentially to form short and strong (linear) hydrogen bonds.

As stated by Bombicz et al. [9] there has been a long-term effort in the field of crystal engineering (and latterly synthonic engineering) to influence or favourably fine tune structural properties by the introduction of substituents or guest molecules of different size, shape and chemical composition to alter the physico-chemical properties of the respective crystals. It is one of the aims of this study to use this knowledge to produce new substances with novel properties.

## Experimental

### Crystallography of triamterene

The most recent search of the CSD using ConQuest version 1.18 resulted in two crystal structures for triamterene with CSD refcodes FITZAJ [3] (*R*_*1*_ of 0.090) and FITZAJ01 [10] (*R*_*1*_ of 0.0739). Since FITZAJ is disordered with some question as to the exact space group and FITZAJ01 is possibly twinned we decided to collect a further dataset using a good quality crystal (CCDC Deposition Number: 1532364, see Additional file 1). For the purpose of comparison, the relevant crystal data for previous studies and this work is shown in Table 1.

**Table 1:** Selected crystallographic data for triamterene

	FITZAJ	FITZAJ01	This work [CCDC: 1532364]
Crystal morphology	Colourless platelets	Yellow block	Yellow block
Data collection temperature (K)	291 (2)	173 (2)	180 (2)
Radiation	Cu (1.54178 Å)	Mo (0.71073 Å)	Cu (1.54178 Å)
Crystal system	Triclinic	Triclinic	Triclinic
Space group	*P*Ī	*P*Ī	*P*Ī
*a* (Å)	7.440 (1)	7.4659 (8)	7.4432 (15)
*b* (Å)	10.164 (1)	10.0257 (12)	9.993 (2)
*c* (Å)	16.666 (2)	16.7147 (19)	16.648 (3)
*α* (°)	77.43 (1)	77.579 (9)	77.55 (2)
*β* (°)	88.75 (1)	87.490 (9)	87.54 (3)
*γ* (°)	88.56 (1)	86.937 (9)	87.09 (3)
Volume (Å^3^)	1229.5	1219.4 (2)	1207.0 (4)
No. of reflections used	4251	4567	4571
No. of observed reflections	3186 [*F*_*o*_ > 3sig*]	3300 [I > 2sig(I)]	3786 [I > 2sig(I)]
*Z, Z′*	4, 2	4, 2	4, 2
*R*_*1*_ factor	0.090	0.0739	0.0360
Calculated density (g/cm^3^)	1.37	1.380	1.394
Packing coefficient	67.8	67.3	68.0

Lath-shaped crystals of triamterene were obtained by dissolving 10 mg of triamterene in 30 ml methanol and dissolution was aided by heating at 50 °C, constant stirring and sonication. After seven days the solution was filtered and allowed to evaporate at room temperature. Triamterene crystallized in the triclinic space group *P*Ī, with *Z* = 4. The crystal chosen for analysis had a minor twin component related to the major component by a twofold rotation around the *a* axis and this was ignored in the integration without any ill effects.

**Fig. 1:**
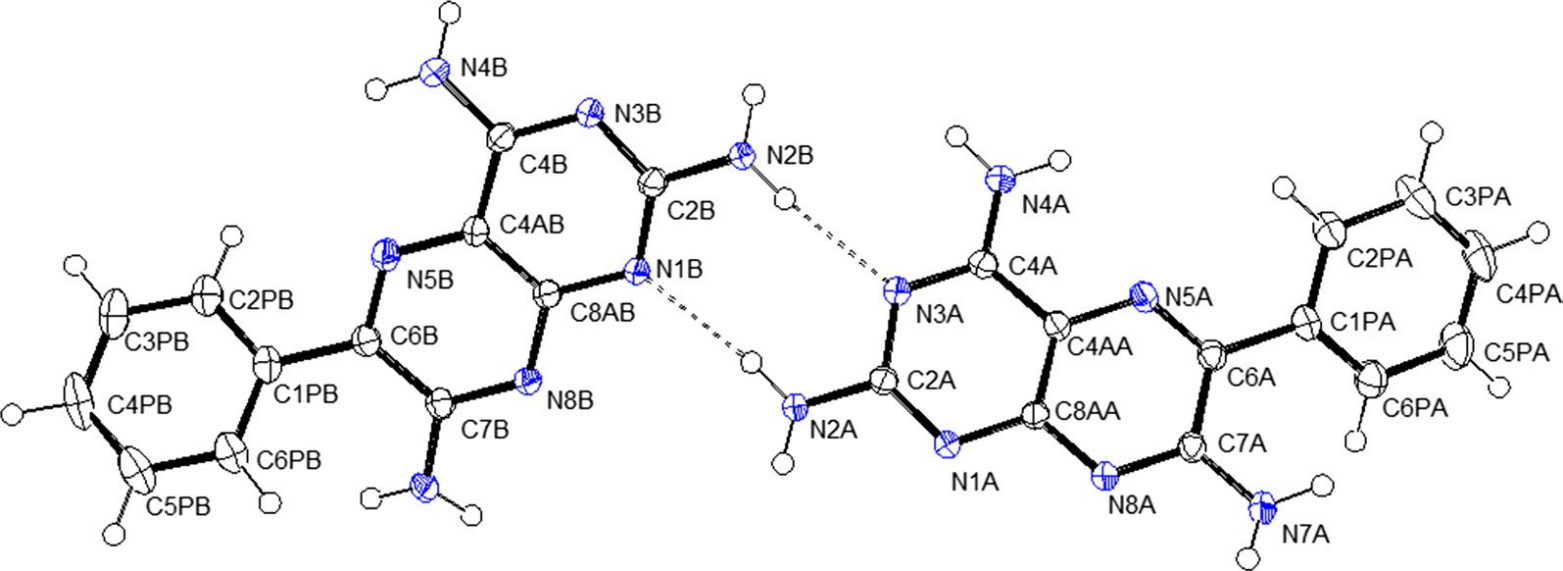
An ORTEP-3 representation (ellipsoids at 50% probability) of the two independent molecules of triamterene that are related by the pseudo-symmetry operation *½* + *x, ½*−*y, ½*−*z* and showing the crystallographic numbering scheme

The independent molecules of triamterene with the crystallographic numbering scheme are shown in the ORTEP 3 for WINDOWS [11] representation in Fig. 1.

The independent molecules may be distinguished by the conformation of the phenyl rings around the single C1P–C6 bond (C2PA–C1PA–C6A–C7A = −143.77 (13)° for molecule A and C2PB–C1PB–C6B–C7B = −147.77 (13)° for molecule B) between the substituted pyrazine and phenyl moieties of the triamterene molecule. This creates a pseudo-chiral configuration at the C6 atom and the action of the crystallographic inversion centre present in space group PĪ produces two sets of enantiomerically related molecules.

The calculated densities and packing coefficients for all three structures published to date (see Table 1) are standard for a closely packed molecular crystal and the absence of polymorphism to date suggests a thermodynamically stable structure.

## Results

### Analysis of hydrogen bonding

Interpretation of the hydrogen bonding in triamterene was carried out using a combination of hydrogen bond connectivity, topology and graph set analysis. This approach is intended to classify hydrogen bonds in a complicated system with a large number of potential donors and acceptors using a simple set of identifiers.

### Numbering scheme

Given the molecular structure of triamterene shown in Scheme 1 it is anticipated that the hydrogen atoms of the 2, 4 and 7 amino groups (H2, H3, H4, H5, H6 and H7) will act as hydrogen bond donors and the pteridine ring nitrogen atoms (N1, N2, N3, N4, N5, N7 and N8) will act as hydrogen bond acceptors in the formation of a hydrogen-bonded crystal structure.

**Scheme 2:**
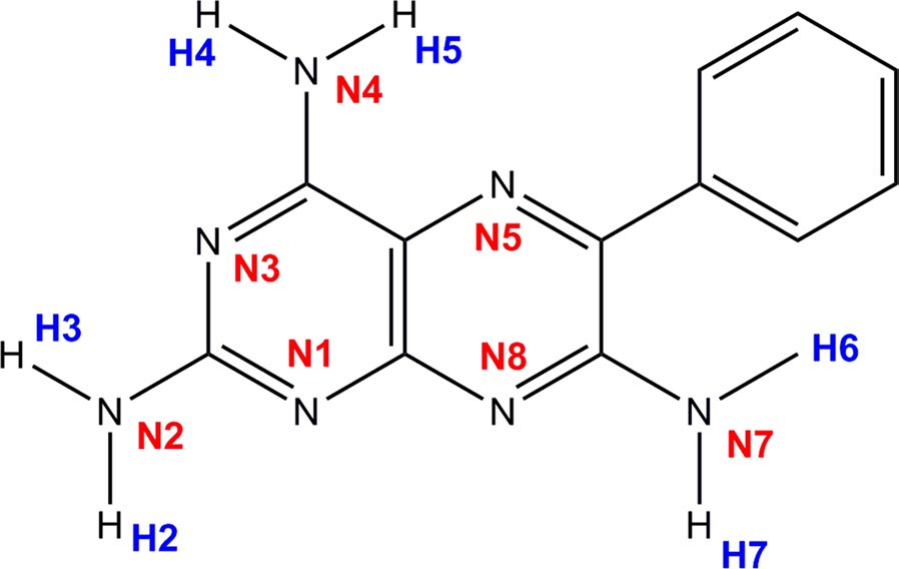
The abbreviated numbering scheme used in this study for triamterene showing all potential hydrogen bond donors and acceptors. All atoms are suffixed by either A or B to allow for identification of the independent molecules of triamterene in subsequent analysis

The numbering scheme we adopt for this study obeys the IUPAC rules for pteridine like molecules and identifies the atomic positions of all ring nitrogen atoms (potential acceptors) and all the hydrogen atoms (potential donors) that may be involved in hydrogen bonding. The numbering scheme is written in accordance with the rules for labelling atoms of the International Union of Crystallography. See Scheme 2 for details.

### Hydrogen bonding in triamterene

Hydrogen bond connectivity and therefore the first stage in defining topology is easily achieved using standard crystallographic software. The traditional approach is to create a list of atom–atom contacts (which immediately identifies the connectivity) together with symmetry operations used to define the contact. The extensive output of the multi-purpose crystallographic tool, PLATON [12] is used throughout this study.

### PLATON terms and notations

Historically, the 555 terminology used in PLATON arose from the Oak Ridge program ORTEP [13]. The original version of ORTEP used a series of instructions (cards) to encode symmetry. Individual atoms were denoted by a 6 component code in which the last 2 digits signify the number of the symmetry operator, the proceeding 3 digits give the lattice translation and the leading digits the atom number. The translation component is such that 555 means no lattice translation. The atom designation ordered by the code [3 654 02], for example, specifies the third atom is transferred by symmetry operation number 2 then translated by [1, 0, −1] along the unit cell vectors.

In the methodology of PLATON connected sets of atoms are assembled by first fixing a suitable atom of the molecule of the greatest molecular weight. A search is then undertaken from this atom in order to identify atoms that are connected to it and this procedure continues from each atom until no new bonded atoms are found. In the simple case of one molecule per asymmetric unit the molecule in the position defined by the position defined by the atom coordinates used in the refinement model is denoted by the identity code 1555.01. Symmetry related molecules are then located and denoted using the general code ***sklm***, where ***s*** is the number of the symmetry operation of the space group (as defined by PLATON) and ***k***, ***l*** and ***m*** the translation components. Such groups of molecules are termed asymmetric residual units (ARUs) in PLATON. It is to be noted that if the position of a molecule coincides with a space group symmetry operation, such as an inversion centre, mirror plane or rotation axis the symmetry operation to generate the symmetry related atoms in the molecule is added to the ARU list. If there is more than one molecule in the asymmetric unit they are each given the suffix .01, .02 etc.

Using this methodology the hydrogen bond connectivity for molecules A and B of triamterene are shown in Table 2. At this stage, it is important to understand that molecule A (MERCURY, crystallographic and graph set terminology) corresponds to residue 1 or .01 (PLATON and topological terminology) and, similarly, molecule B corresponds to residue 2 or .02. With this in mind, Table 2 contains details of D–H…A bonds and angles generated for hydrogen bonds satisfying the default criteria of distance (D…A) being <R(D) + R(A) + 0.50 Å whilst that of (H…A) is <R(H) + R(A) − 0.12 Å and angle (D–H…A) is >100.00; where D is a potential donor, A is a potential acceptor and R is the radius of the designated atom type.

**Table 2:** Hydrogen bonding connectivity in triamterene

No.	Type	Residue	Donor–H…A	[ARU]^a^	D–H	H…A	D…A	D–H…A
1		1	N2A—H2A…N3B	[1655.02]	0.887 (15)	2.167 (15)	3.0430 (17)	169.4 (16)
2		2	N2B—H2B…N3A	[1555.01]	0.920 (16)	2.161 (15)	3.0682 (17)	168.6 (15)
3		1	N2A—H3A…N1B	[1555.02]	0.922 (15)	2.141 (15)	3.0582 (16)	173.1 (14)
4		2	N2B—H3B…N1A	[1455.01]	0.911 (15)	2.138 (15)	3.0436 (16)	172.7 (14)
5		1	N4A—H4A…N8A	[1455.01]	0.92 (2)	2.43 (2)	3.1159 (17)	131.3 (15)
6		2	N4B—H4B…N8B	[1455.02]	0.90 (2)	2.46 (2)	3.1130 (17)	130.4 (14)
7	INTRA	1	N4A—H5A…N5A	[–]	0.921 (18)	2.399 (15)	2.7668 (16)	103.7 (11)
8		1	N4A—H5A…N7A	[1455.01]	0.921 (18)	2.597 (16)	3.1791 (18)	121.7 (12)
9	INTRA	2	N4B—H5B…N5B	[–]	0.916 (18)	2.412 (15)	2.7762 (17)	103.7 (11)
10		1	N7A—H6A…N2B	[2767.02]	0.909 (18)	2.338 (17)	3.0426 (17)	134.3 (14)
11		2	N7B—H6B…N2A	[2776.01]	0.889 (18)	2.323 (18)	3.0323 (17)	136.7 (14)
12		1	N7A—H7A…N8A	[2867.01]	0.905 (16)	2.146 (16)	3.0473 (17)	173.5 (15)
13		2	N7B—H7B…N8B	[2776.02]	0.913 (16)	2.125 (16)	3.0288 (17)	170.1 (15)
14	INTRA	2	C6PB–H6PB…N7B	[–]	0.973 (15)	2.544 (15)	2.9913 (19)	108.0 (11)
15	INTRA	1	C6PA–H6PA…N7A	[–]	0.973 (15)	2.597 (16)	3.0149 (19)	106.1 (11)

^a^Translation of ARU-code to CIF and equivalent position code: [1655.] = [1_655] = 1 + x, y, z, [2776.] = [2_776] = 2 − x, 2 − y, 1 − z, [1455.] = [1_455] = − 1 + x, y, z, [2767.] = [2_767] = 2 − x, 1 − y, 2 − z, [2867.] = [2_867] = 3 − x, 1 − y, 2 − z

Based on the ranking scheme for hydrogen bonds of Steiner [14] the first division of hydrogen bonds (No. 1–13) in Table 2 consist of strong/medium strength “structure forming” hydrogen bonds whilst the second division (No. 14–15) are composed of weaker/longer range interactions. Although the default output is acceptable we will not consider the N4A–H5A…N7A interaction further since it is considered to be too weak (based on H…A criteria) to be “structure forming”. The intramolecular interactions between the different components of the molecule are thought to stabilise conformation. They are among the most important interactions in small and large biological molecules because they require a particular molecular conformation to be formed and, when formed, they confer additional rotational stability to the resulting conformation [15].

### Analysis of hydrogen bonded first coordination sphere

Using the coordinates of donor and acceptor atoms output from PLATON (see Table 2 for details) the connectivity of the first co-ordination shell of triamterene can be determined. In typical organic molecular crystals the connectivity of the molecular co-ordination shell is composed of between ten and fourteen neighbours [16]. The coordination sphere has been extensively investigated by Fillipini [17] and Gavezzotti [18] as a basis for their crystallographic database and computational studies for cases involving Z′ = 1. In the case of triamterene where Z′ = 2 we have developed an alternative approach since an understanding of the coordination sphere is an essential step in determining the topology of this hydrogen bonded system.

**Fig. 2:**
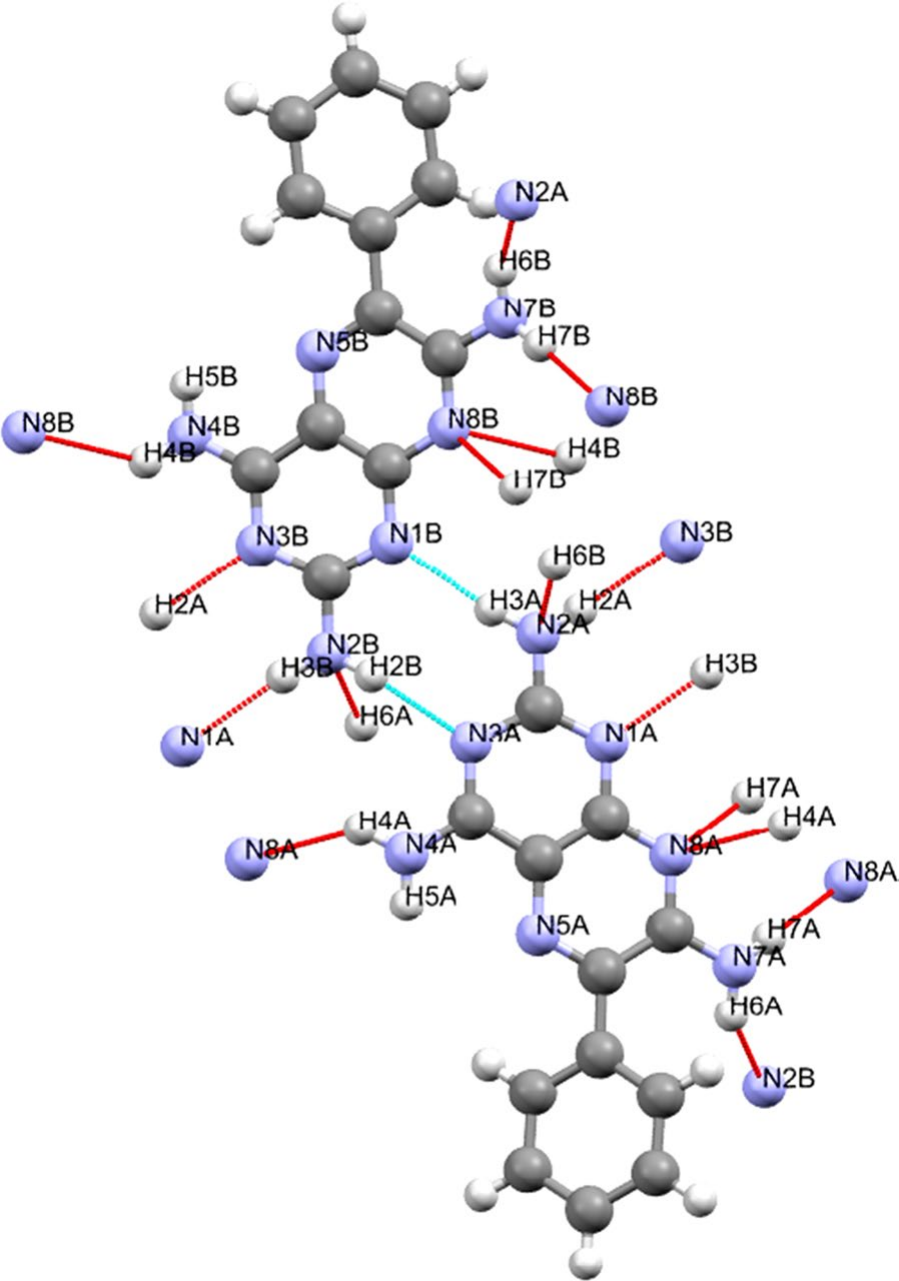
The hydrogen bonded dimer of triamterene

For triamterene, the chemical hydrogen bond connectivity of the first co-ordination sphere may be visualised using MERCURY [19] software to show the hydrogen bonded dimer shown in Fig. 1 and the hydrogen bonded contacts that will form the basis of the next part of the structural discussion (see Fig. 2).

One of the first efforts to classify the different types of hydrogen bonded networks using topological methods was made by Wells in 1962 [20]. He used two parameters for hydrogen bonded systems: the number of hydrogen bonds formed by one molecule he called (n), and the number of molecules to which a given molecule is hydrogen bonded (m). Thus Wells was able to divide hydrogen bonded networks into several classes with the appropriate symbols for *n*_*m*_.

Using a similar scheme Kuleshova and Zorky [21] expanded on this work by classifying hydrogen bonded structures based on the representation of H-aggregates as graphs using homonuclear crystals built up from symmetrically related molecules. Such representation of crystal structures may be described as a graph with topologically equivalent points.

In a recent paper by Shevchenko et al. [22] it is recognised that the coordination sphere significantly affects the topology of the crystal as a whole. A further paper by Zolotarev et al. [23] shows how a study of topology can be incorporated into the prediction of possible crystal forms.

Building on this knowledge, we combine the chemical hydrogen bond connectivity shown in MERCURY (N) with the tabulated topological information provided by PLATON (M) in order to produce the summary seen in Table 3.

**Table 3:** The hydrogen bonded first co-ordination sphere for triamterene to show hydrogen bond connectivity and relevant topological information

1555.01 connected with N hydrogen bonds to/from M ARU(s)							
N	H2A…N3B	N3A…H2B	H4A…N8A	H6A…N2B	H7A…N8A	N8A…H4A	N2A…H6B
	N1A…H3B	H3A…N1B			N8A…H7A		
M	1655.02	1555.02	1455.01	2767.02	2867.01	1655.01	2776.02

1555.02 connected with N hydrogen bonds to/from M ARU(s)							
N	H4B…N8B	N8B…H4B	N3B…H2A	H2B…N3A	H7B…N8B	H6B…N2A	N2B…H6A
			H3B…N1A	N1B…H3A	N8B…H7B		
M	1455.02	1655.02	1455.01	1555.01	2776.02	2776.01	2767.01

From Table 3 the descriptor N:M can be derived using the number of hydrogen bonds (N) connected to the number of molecules to which these hydrogen bonds are attached (M).

### Hydrogen bond connectivity array

As an important step in understanding the crystal structure of triamterene we chose to summarise the combined MERCURY (Fig. 2) and PLATON (Table 3) output discussed above into what we later termed the hydrogen bonding connectivity array. Essentially, each array is a method of representation in which hydrogen bond donors are listed across the vertical columns, for A and B and the hydrogen bond acceptors in horizontal rows in similar fashion. Where a hydrogen bond is encountered the ARU of the contact molecule is entered in the relevant box and the procedure is followed until no more hydrogen bonds are encountered.

The method requires dividing the complete array into smaller regions that may be called ‘zones’. Thus, for a structure with *Z*′ = 2 we can define four zones. Zone 1 (top left) representing any A–A interactions, Zone 2 (top right) for any B–A interactions, Zone 3 (bottom left) for any A–B interactions and Zone 4 (bottom right) for any B–B interactions. The array visualises the co-ordination sphere for each molecule and therefore defines the connectivity of a molecule (node) in the hydrogen bond network. Each node may therefore be given an N:M descriptor where N represents the number of hydrogen bonds and M the number of molecules to which the node is connected.

**Fig. 3:**
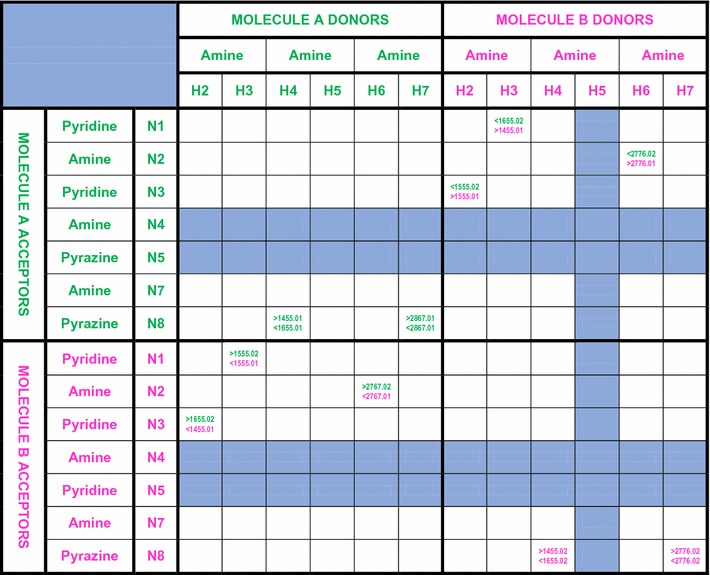
The hydrogen bond connectivity array for triamterene where A and B (coloured *green* and *magenta*) represent the two independent molecules of triamterene, the numerical entries and *directional arrows* represent hydrogen bonds to/from molecules A and B and each entry represents the molecules found in the first coordination sphere. *Areas in blue* do not participate in hydrogen bonding

The hydrogen bond connectivity array for triamterene is presented in Fig. 3.

Thus from the hydrogen bond connectivity array (see Fig. 3) it can be seen that six interactions connect A and B molecules (excluding interactions between molecules A and B) while there are three AA and three BB types. The number of interactions AA, BA, AB and BB represent the number of hydrogen bonds involved and therefore molecule A has a total of ten hydrogen bond connections (entries in green) whilst B also has ten (entries in magenta) which is in agreement with Table 3 above. Topologically, if we consider molecule A and B as centroids then they both have ten hydrogen bonds connected to seven individual molecules (N:M = 10:7). Interestingly, neither of the potential acceptors located at (N5A and N5B) are utilised in hydrogen bonding and this is in good agreement with the pKa data that shows this ring nitrogen to be the least basic but also due to steric hindrance from the phenyl group and the existence of N4–H5…N5 intramolecular bonds from both 4 amino groups. This is in agreement with Etter’s second general rule [24] that states that “[Six-membered-ring] intramolecular bonds form in preference to intermolecular hydrogen bonds”.

A further classification involves grouping the molecules according to their symmetry relationships. From the above analysis and using the PLATON notations four molecules (1455.01, 1655.01, 1655.02 and 1455.02) can be seen to be related to the AB (1555.01 and 1555.02) dimer by translation and five molecules (2867.01, 2767.02, 2776.02, 2776.01 and 2767.01) by a centre of inversion plus translation.

In previous studies by Hursthouse et al. [25] this method of representation yielded valuable symmetry information for comparing the polymorphs of sulfathiazole and sulfapyridine. However, in this instance the chemical (molecular recognition) information provided by the hydrogen bond connectivity array is of primary significance since it will be required for the study of synthon recognition that follows in the subsequent graph set analysis.

This summary agrees well with the information presented in Fig. 2 and Table 3 and is therefore chemically and topologically valid.

### Topology

To understand the extended crystal structure a network approach has been adopted by simplifying the molecules (ARUs) to specified centroids and the hydrogen bond interactions to connectors. To achieve this we again employed the extensive output of PLATON and plotted the hydrogen bond connectivity using orthogonal coordinates by hand. More recently, we have used the program TOPOS [26] to create the overall network representation but we still use the PLATON output to provide very useful topological information.

**Fig. 4:**
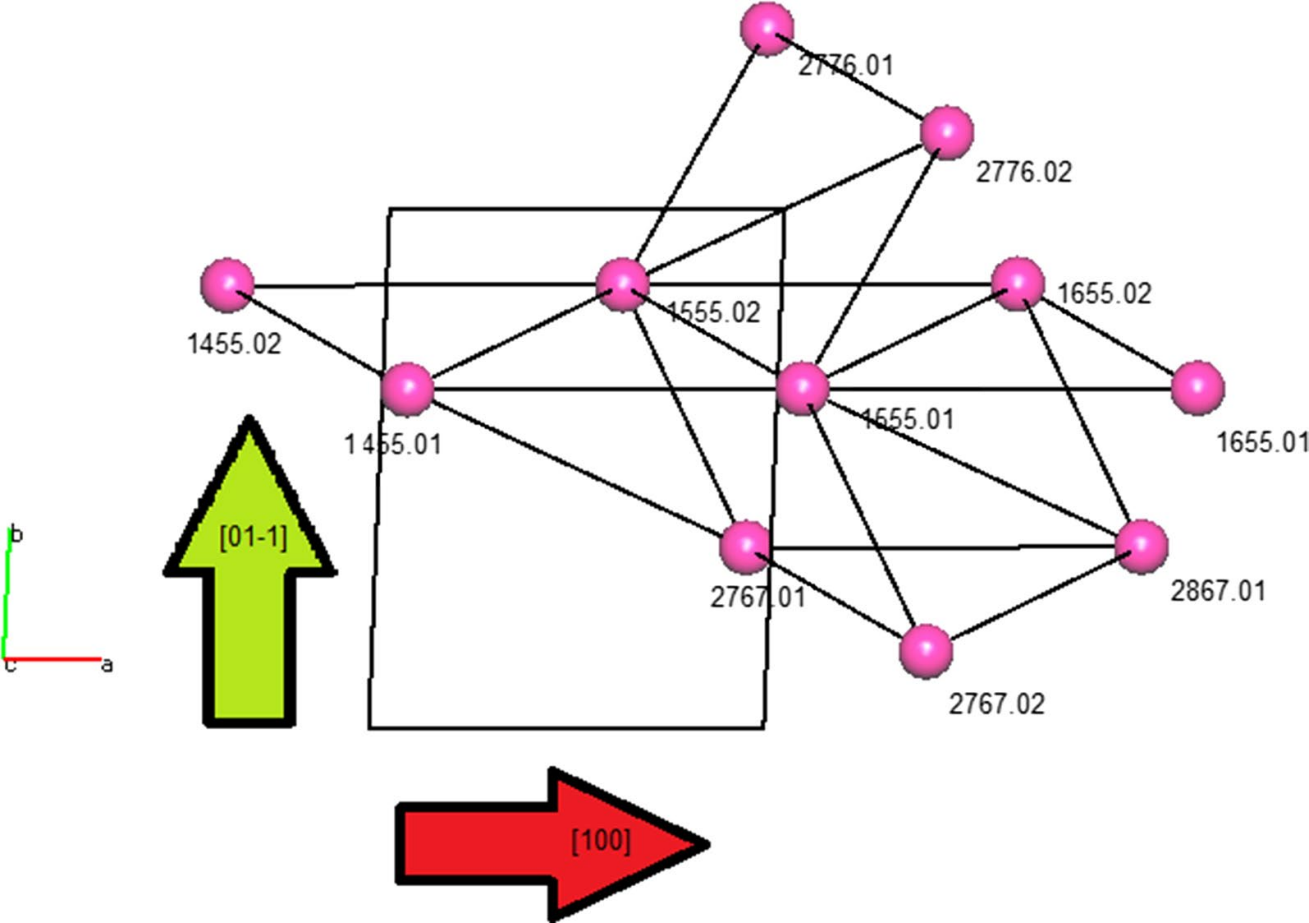
The first coordination sphere of triamterene showing molecules as centroids and hydrogen bonds as connectors with the directions of the base vectors for this system shown using *green* and *red arrows*

Using TOPOS the first coordination sphere (as defined as the nearest hydrogen bond for each A or B molecule of triamterene) can be represented as centroids (molecules) joined by connectors (hydrogen bonds). See Fig. 4.

**Fig. 5:**
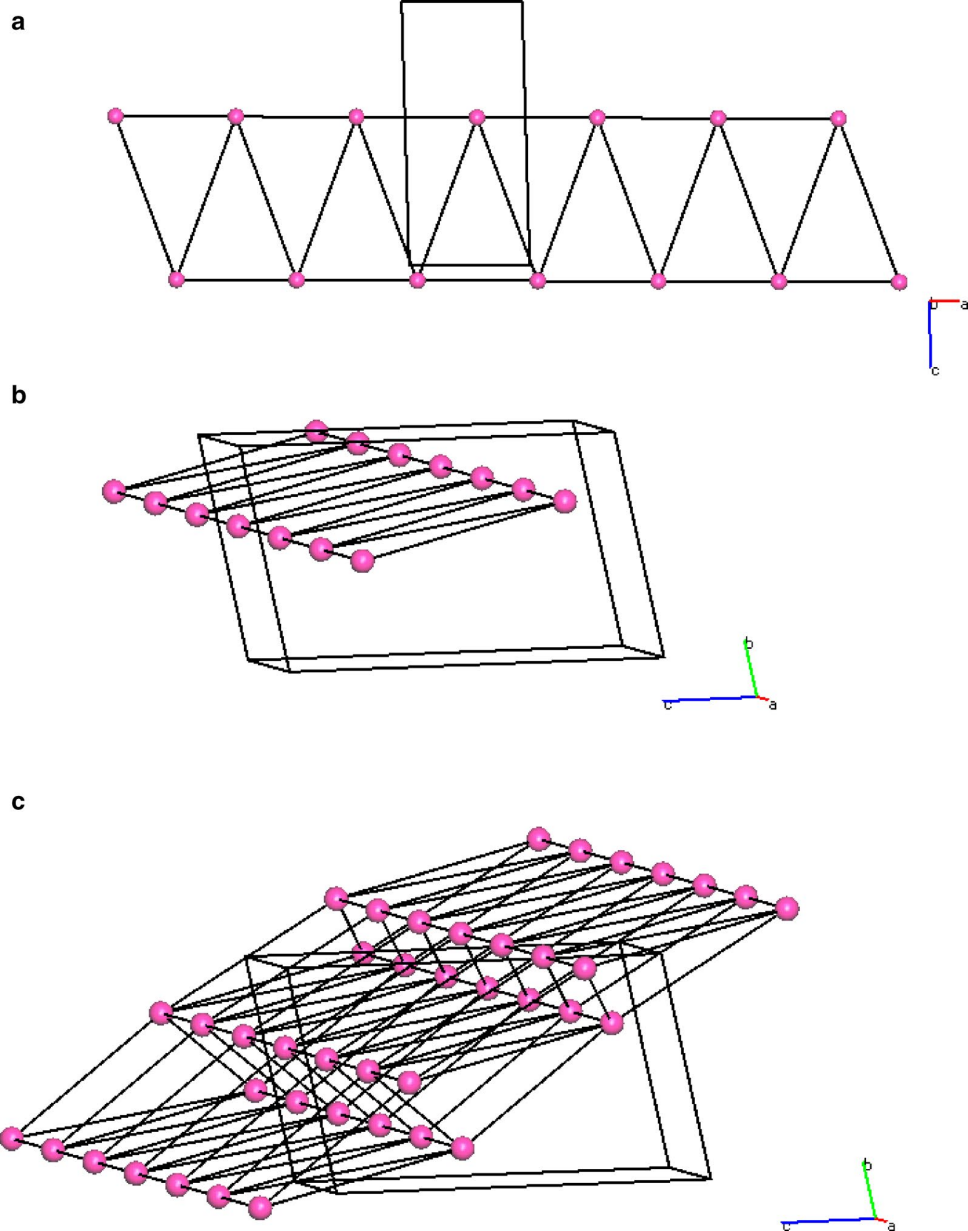
Topology of triamterene showing **a** the AB chain looking down [010], **b** the AB chain viewed down [100] and **c** the full topology of the sheet down (01−1) showing the [100] chain in the same orientation as *(b)* above

Analysis of the ARU data allows for identification of the important topological components of the crystal structure in terms of both directionality and dimension. From Fig. 5 the first coordination sphere is seen to be composed of two essential base vectors [01−1] and [100] (directionality given by green and red arrows respectively) that combine to form a sheet structure in the plane (011).

Now that the essential base vectors have been identified we can start to simplify the structure with a view to understanding the key components in its construction. Essentially, all residues identified by PLATON as being related by translation are approximately planar forming ribbons in the [100] direction whilst those linked by centres of inversion will be out of the plane and link adjacent ribbons in the [01−1] direction (see Fig. 5 for details).

The full topology in Fig. 5 shows the centroids (triamterene molecules) can be described as seven coordinate and the structure extends in two directions [100] and [01−1] to form a sheet in the plane (011). It can be seen from this representation that triamterene is composed of AB ribbons that are connected by hydrogen bonds through centres of inversion to form a 2D sheet.

Due to the shape of the triamterene molecule (long and narrow) and the choice of the centroid as a representation of the molecule some of the out of plane connectors are unrealistically long. Therefore, in order to facilitate the understanding of the topology of the triamterene structure the centroids 2767.02, 2776.01, 2776.02 and 2767.01 are omitted. This is a standard procedure for establishing the essential hydrogen bonded network when using topological methods [27]. The advantages are that this procedure gives a simplified model of the structure whilst retaining the essential topological properties of the hydrogen bonded system. It should be noted at this point that due to this simplification procedure the N:M descriptor for molecules A and B becomes 8:5.

**Fig. 6:**
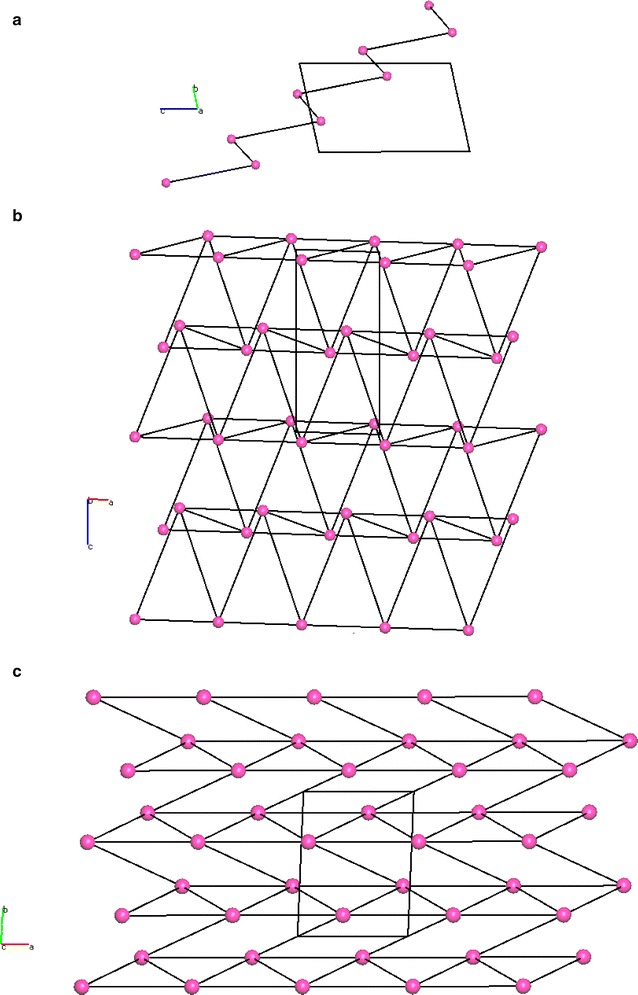
TOPOS representation of the simplified hydrogen bonded network for triamterene showing **a** view down [100], **b** view down [010] and **c** view down [001]. Each molecule is represented as a centroid and hydrogen bonds are shown as connectors

Using TOPOS and PLATON it is now possible to identify the essential hydrogen bonded connections beyond the first coordination sphere and therefore be able to visualise the simplified network structure. See Fig. 6.

It is now be possible to relate the topological ARU information provided in Fig. 6 to the information provided by interpretation of the hydrogen bond chemical connectivity array and subsequent graph set analysis.

At one time graph set analysis would have been completed by visual inspection but owing to the complex nature of the hydrogen-bonded network noted in the triamterene crystal structure, MERCURY software is used to automatically identify the full graph set matrix up to the second level (synthons involving two hydrogen bonds).

### Graph set analysis

In the methodology of Bernstein et al. the repeating hydrogen-bonding motifs are designated by descriptors with the general symbolisation ${\text{G}}_{d}^{a}$(n) where G indicates the motif, namely chains (C), rings (R), intramolecular (S) and discrete (D); *a* and *d* represent the number of acceptors and donors and (n) the number of atoms contained within the motif. Thus, the graph set symbol ${\text{R}}_{2}^{2}$(8) indicates an eight membered ring which contains two donor atoms and two acceptor atoms. For a full explanation of the graph set approach see Bernstein [28].

With atoms identified according to the numbering scheme described in Scheme 2 an abbreviated cif file is created in MERCURY in which the atoms are grouped by residue (molecule A or B) and then used as input for the calculation of the graph sets. This is found to be a necessary extra step in the procedure included to retain continuity and order between the topological and graph set discussions that follow (see Additional file 2).

The unitary graph sets are formed by individual hydrogen bonds whilst the binary graph sets contain up to two different hydrogen bonds. The donors and acceptors associated with independent molecules are designated A and B respectively and for completeness graph sets up to the level 2 are identified with a maximum ring size of six hydrogen bonds, maximum chain size of four hydrogen bonds and a maximum discrete size of four hydrogen bonds for each motif identified.

**Fig. 7:**
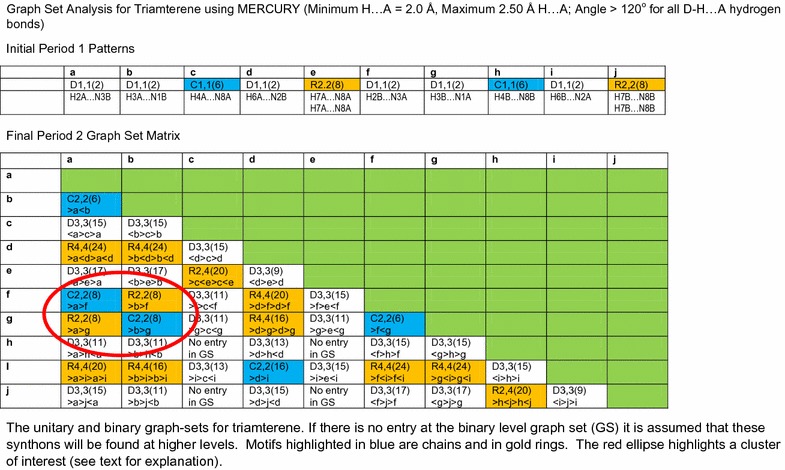
The unitary and binary graph-sets for triamterene. Where there is no entry for the binary level graph set (GS) it is assumed that this synthon will be found at higher levels

For the purposes of the graph set analysis undertaken for triamterene the hydrogen bonds are defined as having a minimum H…A distance = 2.00 Å, and a maximum H…A distance of 2.50 Å with a minimum D–H…A angle of >120° (allowing for correlation with the PLATON intermolecular data presented in Table 2). See Fig. 7 for details.

The unitary graph sets highlight individual hydrogen bonds and show that the two independent molecules have the same unitary motifs whilst the binary graph sets (involving two independent hydrogen bonds) show molecules AA and AB and BB are linked by hydrogen bonds in discrete chain, dimer and ring configurations.

### Synthons found in the crystal structure of triamterene

The hydrogen bonded dimers, rings and chains are highlighted by their graph sets and their relationship explored. Synthons are identified by their graph set descriptor, ${\text{R}}_{d}^{a}$[n] plus a motif identifier (see Fig. 7 for details). This methodology allows for discrimination between synthons that share the same descriptor. In cases where no subscript and/or superscript is shown, one donor and/or one acceptor is implied.

The discussion that follows will describe how the dimer synthons, chain synthons and ring synthons highlighted in Fig. 7 combine to create the crystal structure of triamterene.

Although represented by the same graph set descriptor it is clear that some graph sets involve different positions on the triamterene molecule and therefore are distinguished by the hydrogen bonds used in their creation. These graph sets are termed isographic and discussed in greater detail in the paper by Shimoni et al. [29]. However, for the purposes of this discussion the abbreviated designation of the hydrogen bond type will be used throughout (see Fig. 7 for details) in order to distinguish between isographic systems. So, for example, hydrogen bond H2A…N3B will be referred to as hydrogen bond [a], hydrogen bond H3A…N1B as hydrogen bond [b] etc. See Fig. 7 for the designation of all motifs (hydrogen bonds) used in this system.

**Fig. 8:**
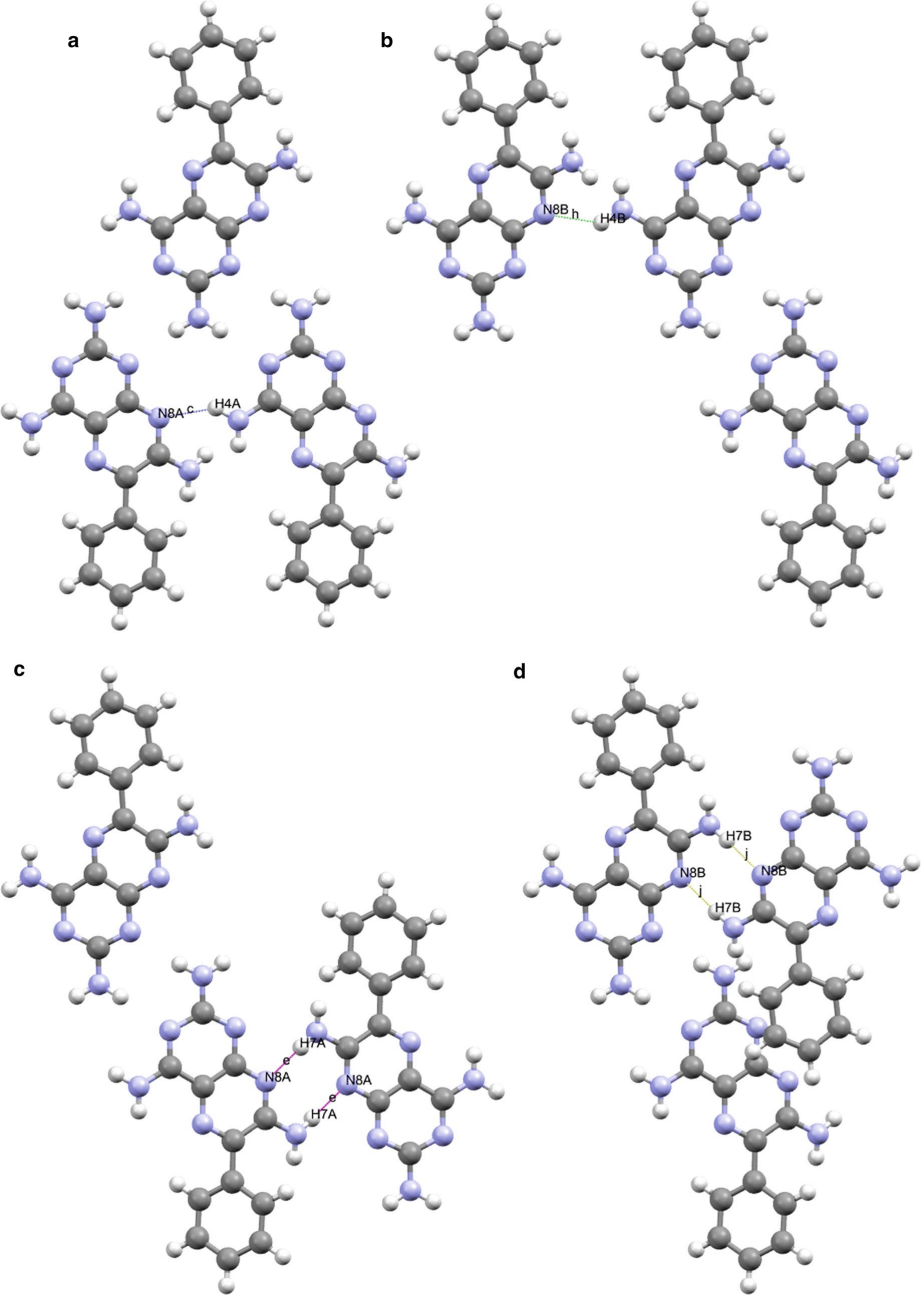
Some examples of structure forming unitary motifs clockwise from **a** C[6]·[c], **b** C[6]·[h], **c** ${\text{R}}_{2}^{2} 8$·[>e>e] and **d** ${\text{R}}_{2}^{2} 8$·[>j>j] all viewed down the *b* axis

Examination of the complete set of unitary motifs for triamterene (see Electronic Supplementary Data (ESI) or Additional file 3: Figure S2 for details) highlights graph sets C[6]·[c] and C(6)·[h] and ${\text{R}}_{2}^{2} 8$·[>e>e] and ${\text{R}}_{2}^{2} 8$·[>j>j]. The graph sets C(6)·[c] and C[6]·[h] show the independent molecules of triamterene exist in separate AA and BB chains linked by H4A…N8A and H4B…N8B hydrogen bonds respectively. Whilst, the graph sets ${\text{R}}_{2}^{2} 8$·[>e>e] and ${\text{R}}_{2}^{2} 8$·[>j>j].show these chains are also linked to adjacent chains by AA and BB dimers containing H7A…N8A and H7B and N8B hydrogen bonds to form homo-dimers These selected motifs are shown in Fig. 8.

**Fig. 9:**
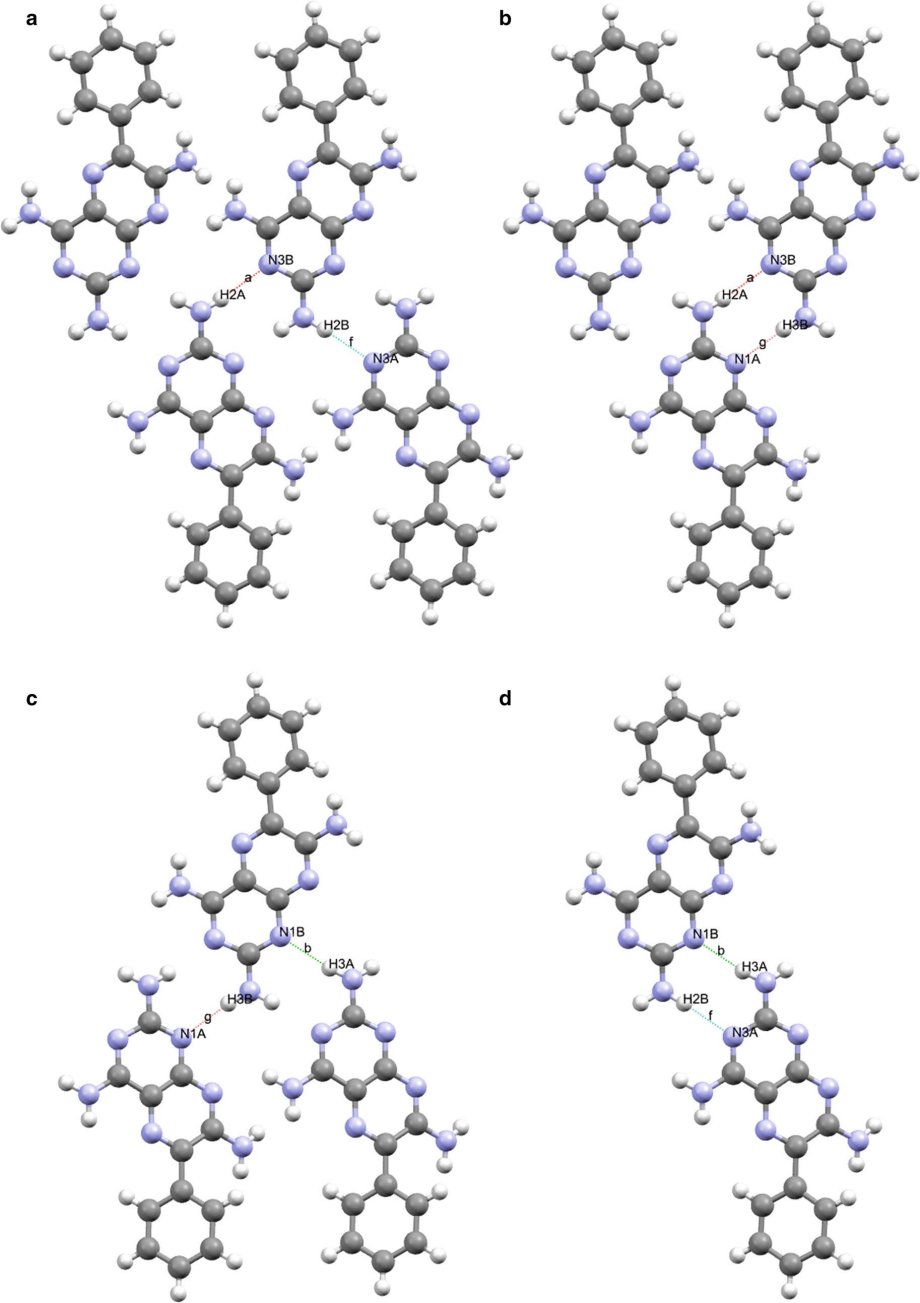
Some examples of structure forming binary synthons clockwise from **a** ${\text{C}}_{2}^{2} 8$·[>a>f], **b** ${\text{R}}_{2}^{2} 8$·[>a>g], **c** ${\text{C}}_{2}^{2} 8$·[>b>g] and **d** ${\text{R}}_{2}^{2} 8$·[>b>f] all viewed down the *b* axis

At the binary level, we begin to see some interesting interactions between the independent molecules (see Fig. 7 and ESI or Additional file 3: Figure S3 for details). There is an interesting cluster (highlighted in red in Fig. 7) involving the interaction between hydrogen bonds [a] (H2A…N3B) and [f] (H2B…N3A) and [a] (H2A…N3B) and [g] (H3B…N1A) to form the ${\text{C}}_{2}^{2} 8$·[>a>f] and ${\text{R}}_{2}^{2} 8$·[>a>g] synthons respectively. In analogous fashion hydrogen bond [b] (H3A…N1B) interacts with [g] (H3B…N1A) and [f] (H2B…N3A) to form ${\text{C}}_{2}^{2} 8$·[>b>g] and ${\text{R}}_{2}^{2} 8$·[>b>f] synthons. These synthons are responsible for completing the ribbon structure that is supported by the C [6] chains described by unitary motifs in the previous section. The ${\text{R}}_{4}^{4} 24$·[>a<d>a<d] and ${\text{R}}_{4}^{4} 24$·[>f<i>f<i] synthons provide a valuable structural role supporting the ${\text{R}}_{2}^{2} 8$ homodimers between ribbons within the sheet (Fig. 9).

To summarise, the tape formed by the binary synthons ${\text{R}}_{2}^{2} 8$·[>a>g] and ${\text{R}}_{2}^{2} 8$·[>b>f] is created using triamterene A and B molecules and creates hydrogen bonded dimers linked by further hydrogen bonded chains with the C[6] unitary motif to form a ribbon. This ribbon is attached to further adjacent ribbons by extending the structure through centrosymmetric dimers ${\text{R}}_{2}^{2} 8$·[>e>e] and ${\text{R}}_{2}^{2} 8$ ·[>j>j] which are supported by the ${\text{R}}_{4}^{4} 24$·[>a<d>a<d] and ${\text{R}}_{4}^{4} 24$·[>f<i>f<i] synthons respectively.

The above discussion forms the basis of our understanding of molecular recognition in the crystal structure of triamterene up to the binary level but a consideration of the topology of the structure can help us discover further graph sets of higher level and, therefore, allow us to identify further structure forming bonds through their topological properties.

As we have seen from our discussion of topology, the hydrogen bonding network can be summarised by a consideration of the first coordination sphere and so by looking at the information contained within this representation we should be able to identify further important factors in the crystal growth of triamterene mediated through hydrogen bonds.

**Fig. 10:**
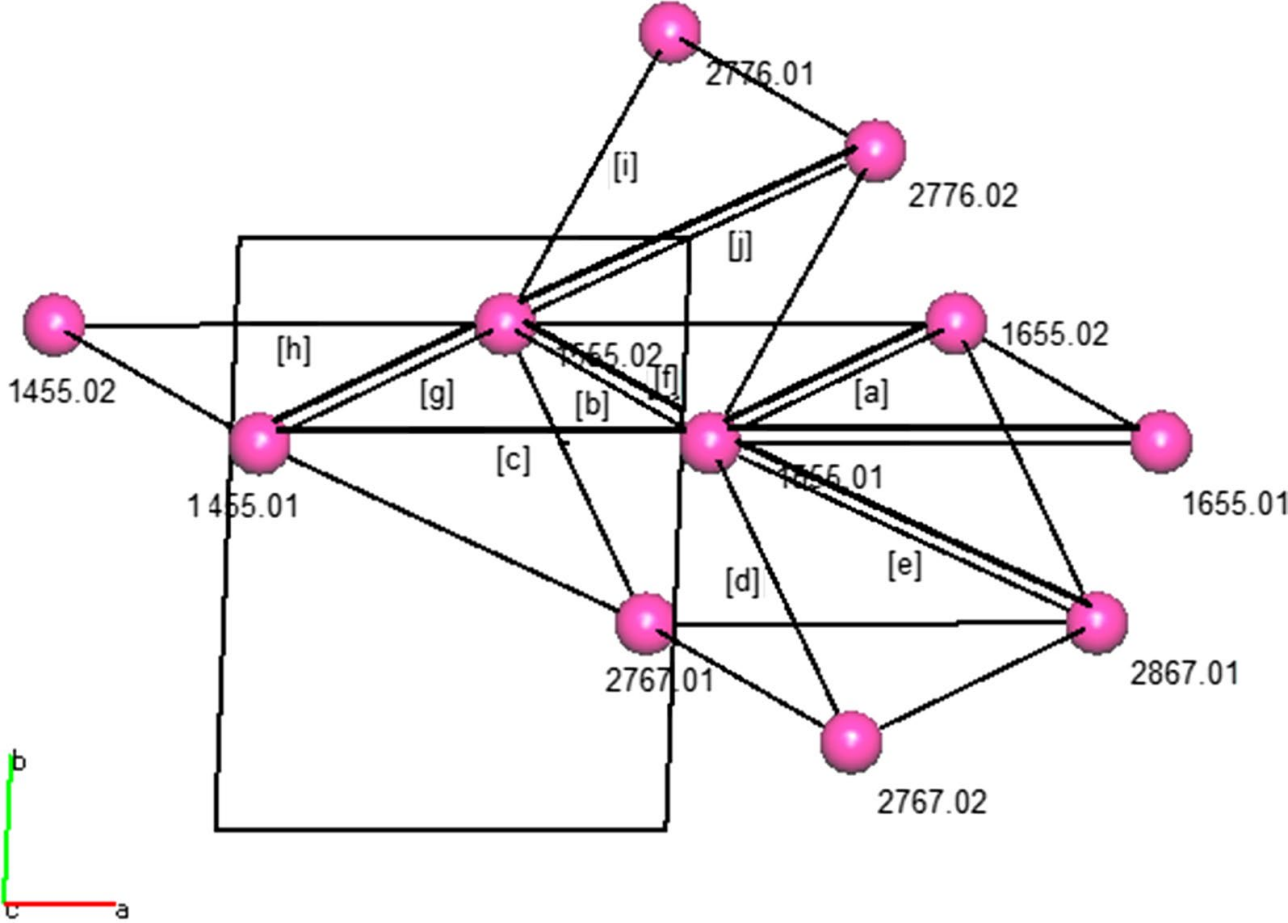
Topology of the first coordination sphere of triamterene to show molecules (centroids), connectors (hydrogen bonds) and designated unitary motifs [*in brackets*] as viewed down [001]. See text for further explanation

The first step of this process is to identify those hydrogen bond motifs that have been highlighted in the discussion of graph sets above. In order to relate the graph set work to the topology all hydrogen bonds are given their graph set designation and molecules are identified using their ARU designator *as per* previous discussions (see Fig. 10 for details).

Using this methodology the complete topology and graph set description can be reduced to one concise representation. Those linkages not labelled in this diagram (indeed, the whole structure) may be deduced by geometry and symmetry, thus reducing a complicated hydrogen bonding network to a simple set of descriptors.

Inspection of Fig. 10 allows us to identify high level graph sets that may be necessary in future work involving potential polymorphism and cocrystal design.

**Fig. 11:**
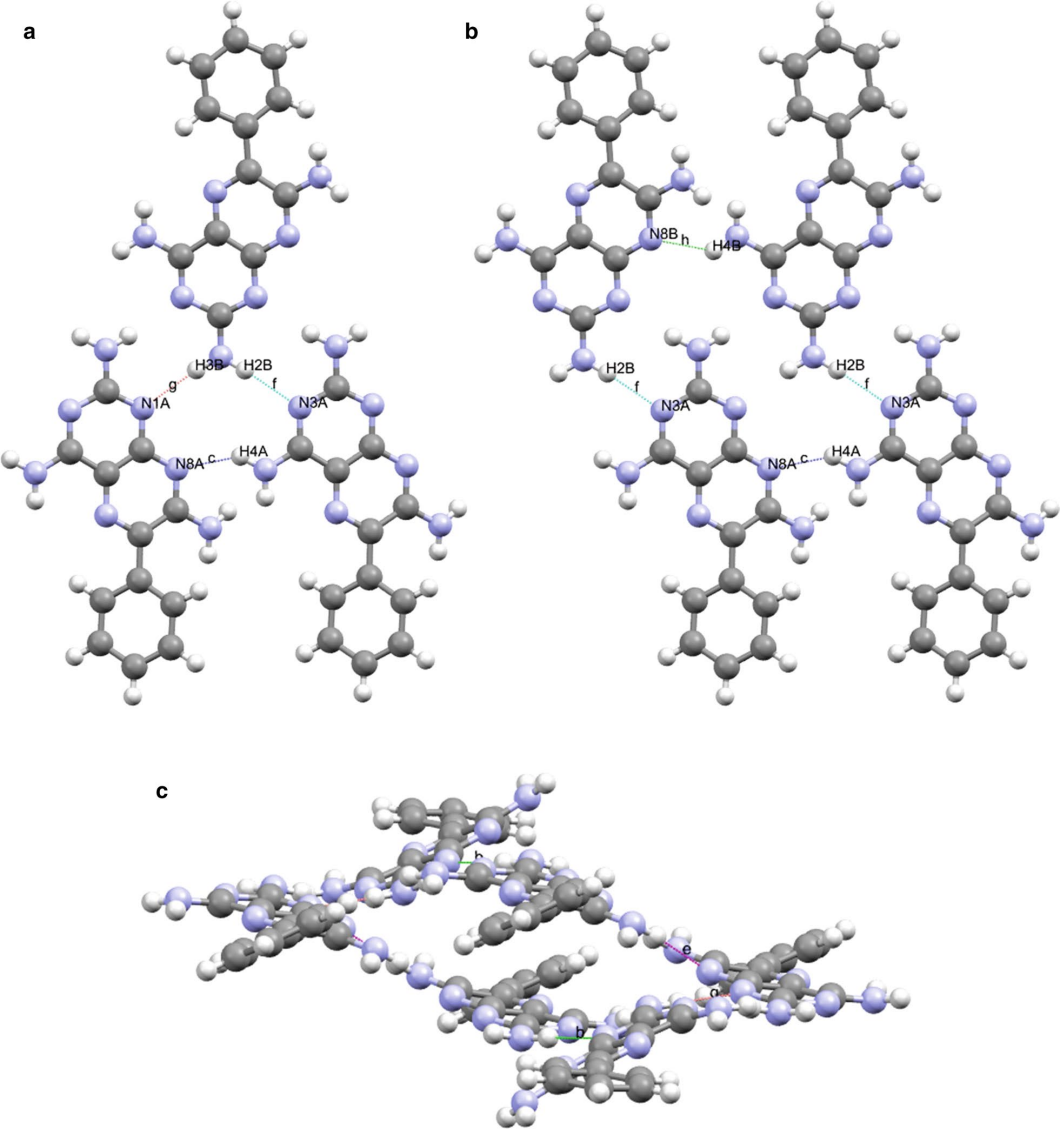
High level graph sets of triamterene clockwise from **a** ${\text{R}}_{3}^{3} 10$·[>c<g>f] viewed down the *b* axis, **b** ${\text{R}}_{4}^{4} 22$·[>c<f<h>f] viewed down the *b* axis and **c** ${\text{R}}_{6}^{6} 32$·[>b>g<e>b>g<e] viewed down the *c* axis

Thus, using a combination of topology and graph set analysis summarised in the graphical representation shown in Fig. 11, the following high level graph sets can be identified:

- The tertiary graph set ${\text{R}}_{3}^{3} 10$·[>c<g>f] is noted between 3 molecules, 1555.01, 1455.01, 1555.02 and 1555.01.
- The tertiary graph set ${\text{R}}_{4}^{4} 22$·[>c<f<h>f] is noted between 4 molecules 1555.01, 1455.01, 1455.02, 1555.02 and 1555.01.
- The tertiary graph set ${\text{R}}_{6}^{6} 32$·[>b>g<e>b>g<e] is noted between 6 molecules 1555.01, 1555.02, 1455.01, 2767.01, 2767.02, 2867.01 and 1555.01.

Figure 11 highlights the synthons found using this method.

Table 4 summarises the selected synthons found during this study of the crystal structure of triamterene.

**Table 4:** Summary of selected hydrogen bond motifs and synthons found in triamterene

Hydrogen bond(s)	Number of molecules	Topology	Graph set descriptor
H4A…N8A	2	1555.01 and 1455.01	C[6]·[c]
H4B…N8B	2	1555.02 and 1455.02	C[6]·[h]
H7A…N8A and N7A…N8A	2	1555.01, 2867.01 and 1555.01	${\text{R}}_{2}^{2} 8$·[>e>e]
H7B…N8B and N8B…H7B	2	1555.02, 2776.02 and 1555.02	${\text{R}}_{2}^{2} 8$·[>j>j]
H2A…N3B and H2B…N3A	3	1555.01, 1655.02 and 1655.01	${\text{C}}_{2}^{2} 8$·[>a>f]
H2A…N3B and H3B…N1A	2	1555.01, 1655.02 and 1555.01	${\text{R}}_{2}^{2} 8$·[>a>g]
H3A…N1B and H3B…N1A	3	1555.01, 1555.02 and 1455.01	${\text{C}}_{2}^{2} 8$·[>b>g]
H3A…N1B and H2B…N3A	2	1555.01, 1555.02 and 1555.01	${\text{R}}_{2}^{2} 8$·[>b>f]
H4A…N8A, N1A…H3B and H4A…N8A	3	1555.01, 1455.01, 1555.02 and 1555.01	${\text{R}}_{3}^{3} 10$·[>c<g>f]
H4A…N8A, N3A…H2B, N8B…H4B and H2B…N3A	4	1555.01, 1455.01, 1455.02, 1555.02 and 1555.01	${\text{R}}_{4}^{4} 22$·[>c<f<h>f]
H3A…N1B, H3B…N1A, N8A…H7A, H3A…N1B, H3B…N1A and N8A…H7A	6	1555.01, 1555.02, 1455.01, 2767.01, 2767.02, 2867.01 and 1555.01	${\text{R}}_{6}^{6} 32$·[>b>g<e>b>g<e]

Further analysis involving the salts and cocrystals of triamterene will allow for identification of the preferred molecular packing unit by comparing the synthons formed in these crystal structures with those found in triamterene. It is anticipated that the structural differences and similarities found between triamterene and the cocrystals will arise from both the ways the sheets are constructed and from their packing sequences. Using this approach it is intended to use a series of dicarboxylic acids to inform our choice of potential API and GRAS coformers and to test this hypothesis using pharmaceutically acceptable examples. According to Bernstein [30], the chemically interesting or topologically characteristic patterns of a system will often appear when more than one type of hydrogen bond is included in the description, hence, the consideration of a range of coformers will be of particular interest in this context.

Since we are now in possession of all the requisite crystallographic, topological and molecular recognition data we can now proceed to discuss the crystal structure of triamterene in terms of crystallography, topology and graph set analysis.

## Conclusions

### Hydrogen bonded dimers, chains, ribbons and sheets

The triamterene molecule exists in the neutral state in the crystal structure of the pure polymorphic form. The molecule has six hydrogen and seven nitrogen atoms that can potentially take part in hydrogen bonding. From our discussions (see “Introduction”), when considering the neutral molecule, the ring nitrogen atom N1 is the obvious choice for best acceptor. In the known repeated crystal structures of the pure phase of triamterene they all have two molecules in the asymmetric unit and all occupy the space group *P*Ī. For the purposes of the following discussion hydrogen bonds are designated according to the scheme shown in Fig. 7.

**Fig. 12:**
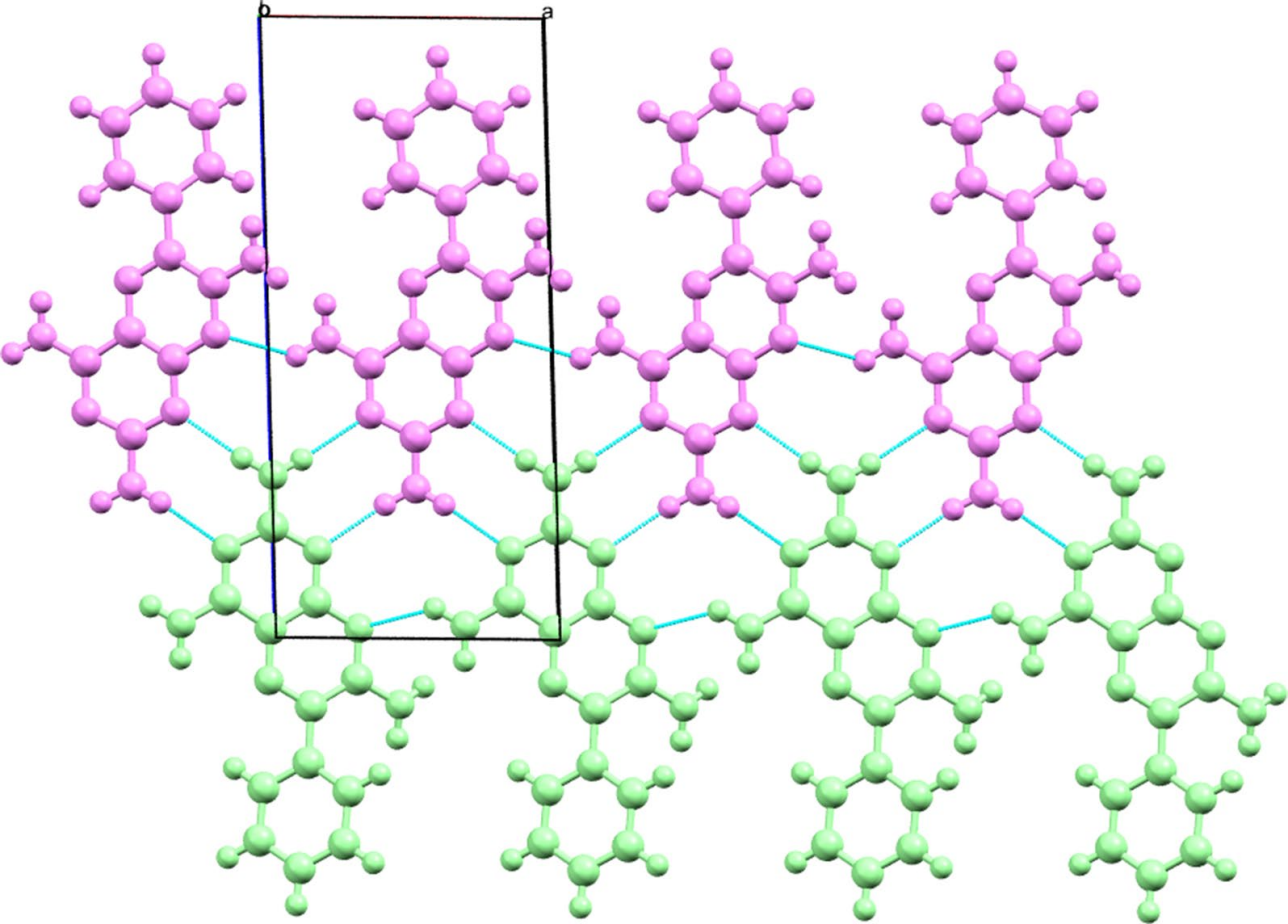
Part of the hydrogen bonded network of triamterene showing the ribbons formed between A (*green*) and B (*magenta*) molecules as viewed down the *b* direction

The hydrogen bonded dimer (shown in Fig. 2) formed between the independent molecules of A and B made up of H2B of the 2 amino group and the N1B of the pyrimidine ring of a B molecule is linked by a pseudo inversion centre to the N3A and H3A of the 2 amino group of a neighbouring A molecule, thus forming a synthon with the graph set symbol, ${\text{R}}_{2}^{2} 8$·[>b>f]. The A molecule of the dimer is extended by hydrogen bonding in both lateral directions [−100] and [100] directions using hydrogen bonds H2B…N3A and H3B…N1A to form an infinite chain described by the binary graph set symbol, ${\text{C}}_{2}^{2} [6]$·[>f<g] as shown in Fig. 12. In a similar fashion B molecules extend along the [100] axis to form a tape. Additionally, the N8A of molecule A and the 4 amino groups of an adjacent A molecule use one of their protons (H4A) to support the formation of a ribbon by creating a further C[6] chain between adjacent A molecules. In the same way B molecules extend the ribbon by forming a further C[6] chain between translated B molecules. Combining the above motifs and synthons effectively produces a complex hydrogen-bonded four-component ribbon synthon described by the tertiary graph set symbol, ${\text{R}}_{4}^{4} 22$·[>c<f<h>f] and this synthon is repeated by translation along the [100] direction.

**Fig. 13:**
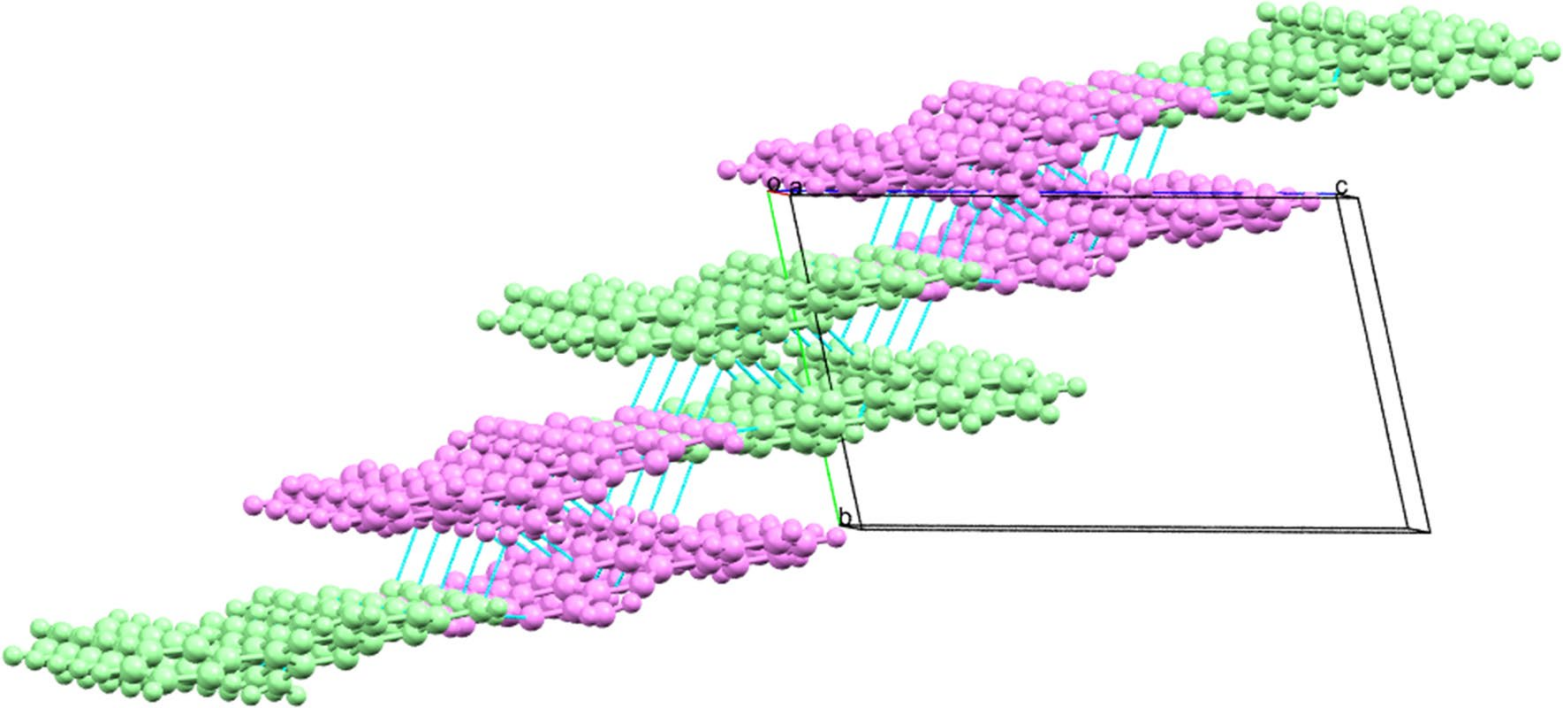
The structure of triamterene showing the relationship between ribbons along [100] and the extension of the structure along [01−1] to produce a hydrogen bonded sheet in the plane (011)

Since each pseudo-symmetric hydrogen bonded AB dimer is finite in the [001] direction due to the hydrophobic nature of the aromatic end groups (effectively blocking growth by hydrogen bonds) other ways are needed to extend the structure if a sheet is to be formed. In the topology of the triamterene structure hydrogen bonds in the [01−1] direction are noted as being structure forming due to the formation of strong centrosymmetric ${\text{R}}_{2}^{2}$(8)·[>e>e] dimers found between the hydrogen H7A of the 7 amino group of an A molecule and the N8A of the pyrazine ring of the molecule immediately below and to the side. In a similar fashion the B molecules also form strong centrosymmetric ${\text{R}}_{2}^{2}$(8)·[>j>j] dimers between adjacent ribbons. Effectively, this strong centrosymmetric dimer alternates between AA and BB molecules in a stepped fashion through the structure and thus allowing growth in the [01−1] direction as demonstrated in Fig. 13.

**Fig. 14:**
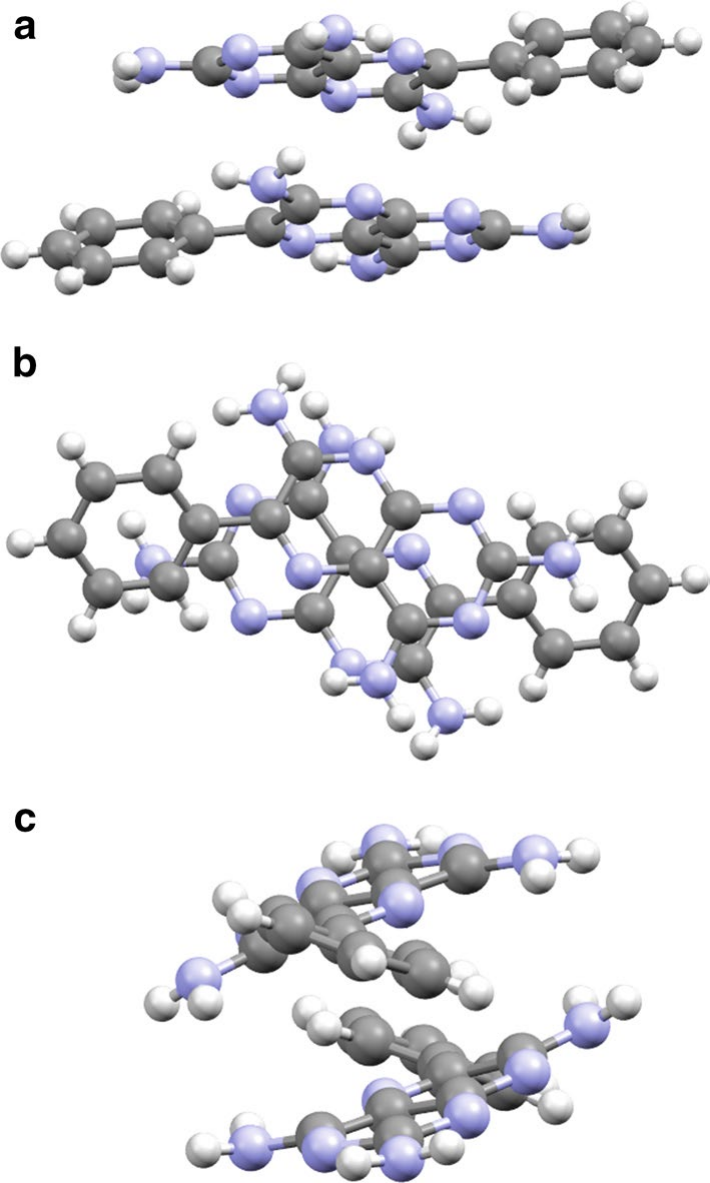
The offset dimer viewed along **a** [100], **b** [010] and **c** [001] that creates the important centrosymmetric synthon that allows the planar π donors and acceptors to form the overlapping sheet structure seen in triamterene

The above structural discussion is based on hydrogen bonding being used to create sheets in two dimensions. It should be noted, however, that there is also a significant interaction within the sheet due to the offset π…π dimers. This interaction involves stacking of pteridine rings of like kind (AA and BB molecules) around centres of inversion at approximate van der Waals separation (~3.5 Å) creating the robust supramolecular synthon seen in Fig. 14. It is this interaction in conjunction with the strong hydrogen bonds described above that are responsible for the stepped nature of the sheet.

Finally, van der Waals forces are responsible for the packing of these sheets in the crystal structure and this completes the full description of the molecular packing found in triamterene.

In summary, the crystal structure of triamterene can be thought of being composed of hydrogen bonded ribbons running in the [100] direction. These are joined by π…π centrosymmetric dimers above and below the plane of the ribbon to allow extension of the hydrogen bonded structure in the [01−1] direction. Combining these structural components creates a stepped sheet in the plane (011). Adjacent terraced hydrogen bonded sheets pack above and below this sheet using van der Waals forces to form the full 3D crystal structure.

## Further work

We hope to be able to use this protocol to study further solid forms with a view to creating optimum physical properties for future applications. Some of the areas of current interest include the study of synthons in solution to determine mechanisms for crystal growth, the study of lattice energy to predict crystal morphology and a study of the polymorphism of pteridine like compounds using the Cambridge Structural Database.

## Additional files

**Additional file 1.** The CIF file (CCDC deposition number: 1532364) for triamterene.

**Additional file 2.** An abbreviated CIF file for triamterene, suitable for input to graph set analysis using MERCURY.

**Additional file 3.** Details of the crystal structure determination, topology (using PLATON and TOPOS) and graph set analysis (using MERCURY).

## Abbreviations

A: hydrogen bond acceptor
ARU: Asymmetric Residual Unit
D: hydrogen bond donor
DHFR: dihydrofolate reductase
DMF: dimethylformamide
DMSO: dimethyl sulfoxide
N:M: Number of hydrogen bonds (N) connected to number of molecules (M)
