# Supplementary material for: Using crystallography, topology and graph set analysis for the description of the hydrogen bond network of triamterene: a rational approach to solid form selection
Source: Chem Cent J. 2017 Jul 13;11:63. doi: 10.1186/s13065-017-0293-1 (PMC5509571; doi:10.1186/s13065-017-0293-1)
Supplement: Supplementary file 3 — Additional file 3. Details of the crystal structure determination, topology (using PLATON and TOPOS) and graph set analysis (using MERCURY). [file 13065_2017_293_MOESM3_ESM.docx]

Using crystallography, topology and graph set analysis for the description of the hydrogen bond network of triamterene: A rational approach to solid form selection

_______________

ELECTRONIC SUPPLEMENTARY DATA

_______________

**Authors**

David S Hughes^1*^

Amit Delori^2^

Abida Rehman^1^

William Jones^1^

**Affiliations**

^1^Department of Chemistry, University of Cambridge, Lensfield Road, Cambridge CB2 1EW, UK

^2^Strathclyde Institute of Pharmacy and Biomedical Sciences (SIPBS), University of Strathclyde, 161 Cathedral Street, Glasgow G4 0RE, UK

Corresponding author

*Department of Chemistry, Lensfield Road, Cambridge CB2 1EW, UK. Email: [dh536@cam.ac.uk](mailto:dh536@cam.ac.uk); Fax: +44 (0)1223 336465; Tel: +44 (0)1223 336468

**Contents**

**1. Crystal structure 3**

**2. Topology using PLATON and TOPOS 4**

**3. Graph set analysis using MERCURY 8**

**1. Crystal Structure**

**Table S1**. Selected Crystallographic data for triamterene.

|  | **Triamterene** |
| --- | --- |
| **Formula** | C_12_H_11_N_7_ |
| **Mr** | 253.28 |
| **Crystal habit** | block |
| **Crystal colour** | yellow |
| **Crystal system** | triclinic |
| **Space group** | *P*Ī |
| ***a*(Å)** | 7.4432(15) |
| ***b*(Å)** | 9.993(2) |
| ***c*(Å)** | 16.648(3) |
| **α(°)** | 77.55(2) |
| **β(°)** | 87.54(3) |
| **γ(°)** | 87.09(3) |
| ***V*(Å^-3^)** | 1207.0(4) |
| ***Z*** | 4 |
| ***D*_calc_(gcm^-1^)** | 1.394 |
| ***T*(K)** | 180 |
| ***λ*Kα (Cu)** | 1.54178 |
| ***μ*(mm^-1^)** | 0.761 |
| **2*θ* range(°)** | 67.416 |
| **Limiting indices** | -8 ≤ *h* ≤ 9  -12 ≤ *k* ≤ 12  -20 ≤ *l ≤* 20 |
| ***F*(000)** | 528.0 |
| **No. of reflections measured** | 31347 |
| **No. of reflns. used** | 4571 |
| **No. unique reflns.** | 3786 |
| **No. of parameters** | 432 |
| **GOF on *F*^2^** | 1.032 |
| ***R*_1_ [*I* > 2*σ*(*I*)]** | 0.0360 |
| **w*R*_2_** | 0.0917 |
| **Final diff. Fourier map (e^-^ Å^-3^) max., min.** | 0.248 and  -0.187 |
| **CCDC deposition number** | 1532364 |

**2. Topology using PLATON and TOPOS**

**PLATON**

**Method**

The wj_b2_0018.cif file is read into PLATON and the output platon.lst contains the information seen above. The space group highlights the Hermann-Mauguin (HM) and Hall notations, together with lattice type and multiplicity. The unit cell contents are listed as centroids or residues (res), sites as fractional coordinates (X, Y and Z), molecular weight (MW), site occupancy factor (SOF), Z (number of molecules in unit cell), and atoms that constitute the asymmetric unit (C is carbon, H is hydrogen and N is nitrogen). Each unit cell is then linked by nearest neighbours to form Aggregate 1 where N is the number of hydrogen bonds and M is the number of molecules to which those hydrogen bonds are linked. The asymmetric residual unit (ARU) represents one molecule and the nomenclature for the ARU coding is described in the manuscript text. Using linear algebra the Aggregate information is analysed in terms of residue, hydrogen bond network network) and base vectors that define the overall topology (plane in this case).

**Results**

Input file: wj_b2_0018.cif

Output file: platon.lst

Summary of PLATON output from platon.lst

Space group symmetry

| Space Group (HM) | Space Group (Hall) | Lattice Type | Triclinic |
| --- | --- | --- | --- |
| P-1 (Laue: -1) | -P1 [Schoenfiles Ci^1] | aP, centric | Multiplicity 2(1) No. 2 |

Symmetry operation(s)

| 1 | 2 |
| --- | --- |
| X, Y, Z | -X, -Y, -Z |

Unit cell contents

| Resd | Site | X(cen) | Y(cen) | Z(cen) | MW | SOF | Z | C | H | N |
| --- | --- | --- | --- | --- | --- | --- | --- | --- | --- | --- |
| 1 | 1 | 1.063 | 0.653 | 1.058 | 253.28 | 1 | 2 | 12 | 11 | 7 |
| 2 | 1 | 0.597 | 0.854 | 0.439 | 253.28 | 1 | 2 | 12 | 11 | 7 |

Aggregate 1

| N:M | ARU | Connected with N hydrogen bonds to from M ARU(S) | | | | | | |
| --- | --- | --- | --- | --- | --- | --- | --- | --- |
| 12:7 | 1555.01 | 1655.02 | 1555.02T | 1455.01T | 2767.02 | 2867.01 | 1655.01T | 2776.02T |
| 10:7 | 1655.02 | 1655.01T | 1555.01 | 1555.02T | 2876.01T | 2876.02T | 1755.02T | 2867.01 |
| 10:7 | 2767.02 | 2767.01T | 2867.01 | 2867.02T | 1546.01T | 1546.02T | 2667.02T | 1555.01 |
| 12:7 | 2867.01 | 2767.02 | 2867.02T | 2967.01T | 1655.02 | 1555.01 | 2767.01T | 1646.02T |

T = Translated molecule (infinite chain etc.)

Analysis of hydrogen bonded aggregate type3 polymeric structure(s)

| Residue | Network | Base Vectors | Plane |
| --- | --- | --- | --- |
| Resd 0 | Infinite (Type 3) 2D network | #1 = [100]  #2 = [01-1] | Plane (011) |

**TOPOS**

**Method**

The wj_b2_0018.cif file is read into TOPOS and analysed using the automatic coordination (AutoCN) module using the method of molecular Voronoi polyhedra to produce an unit cell containing the centroids defined as scandium (Sc) atoms, their number (for labelling purposes to distinguish between centroids), the degree of oxidation (DegOx, not relevant for organic molecules), point group symmetry (Ci), fractional coordinates (X, Y and Z), site occupancy (S) and coordination number (CN). Using the Voronoi model an adjacency matrix is produced of nearest neighbours for molecule (centroid) 1 and molecule (centroid) 2. The adjacency matrix allows for definition of the centroid (name and number), symmetry operator, direction (vector), solid angle (SA), radius or distance (R) and multiplicity (M). Once the adjacency matrix has been defined the topology can be drawn in conjunction with the information on ARUs provided by PLATON.

**Results**

TOPOS input: wj_b2_0018.cif

TOPOS output: crystal data file produced by AutoCN

Atoms

| Name | No. | DegOx | 2,Ci | X | Y | Z | S | CN |
| --- | --- | --- | --- | --- | --- | --- | --- | --- |
| Sc | 1 | 0 | 2i, C1 | 0.06287 | 0.65294 | 0.05764 | 1 | 7 |
| Sc | 2 | 0 | 2i, C1 | 0.59717 | 0.85413 | 0.43871 | 1 | 7 |

Adjacency matrix

Sc1 [CN = 7, Rsd = 0.000] V

| Name & Number | Symmetry | Vector | SA | R | Mult |
| --- | --- | --- | --- | --- | --- |
| Sc1 | -x, -y, -z | 0, 0, -1 | 22.6 | 7.403 | 1V |
| Sc1 |  | 1, 0, 0 | 9.4 | 7.443 | 2V |
| Sc1 |  | -1, 0, 0 | 9.4 | 7.443 | 2V |
| Sc2 | -x, -y, -z | 0, 1, -1 | 5.6 | 8.993 | 2V |
| Sc2 | -x, -y, -z |  | 5.8 | 9.197 | 2V |
| Sc2 |  | 0, 0, -1 | 23.6 | 10.695 | 2V |
| Sc2 |  | -1, 0, -1 | 23.7 | 10.754 | 2V |

Sc2 [CN = 7, Rsd = 0.000] V

| Name & Number | Symmetry | Vector | SA | R | Mult |
| --- | --- | --- | --- | --- | --- |
| Sc2 | -x, -y, -z | 1, 1, 0 | 22.8 | 7.394 | 1V |
| Sc2 |  | 1, 0, 0 | 9.3 | 7.443 | 2V |
| Sc2 |  | -1, 0, 0 | 9.3 | 7.443 | 2V |
| Sc1 | -x, -y, -z | 0, 1, -1 | 5.6 | 8.993 | 2V |
| Sc1 | -x, -y, -z |  | 5.7 | 9.197 | 2V |
| Sc1 |  | 0, 0, 1 | 23.5 | 10.695 | 2V |
| Sc1 |  | 1, 0, 1 | 23.7 | 10.754 | 2V |


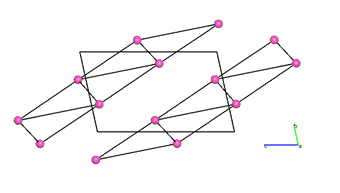


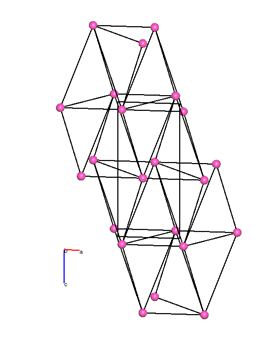


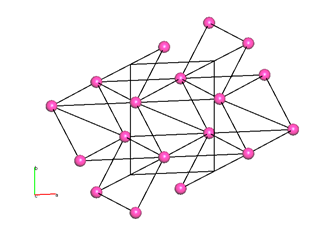


**Figure S1**. TOPOS representation of hydrogen bond connectivity for triamterene showing a) view down [100], b) view down [010] and c) view down [001].

For further information on the workings of TOPOS the interested reader is referred to the TOPOSPro website <<http://topospro.com>> where a worked example involving the topological analysis of the crystal structures of tranexamic acid derivatives is available.

**3. Graph set analysis using MERCURY**

| D1,1(2)•[a] (H2A…N3B) | D1,1(2)•[b] (H3A…N1B) |
| --- | --- |
| 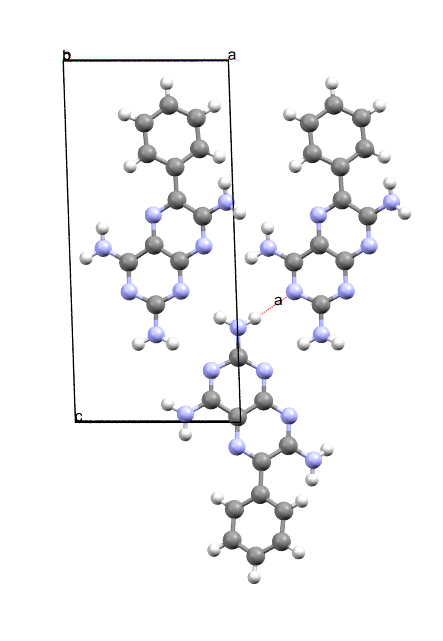 | 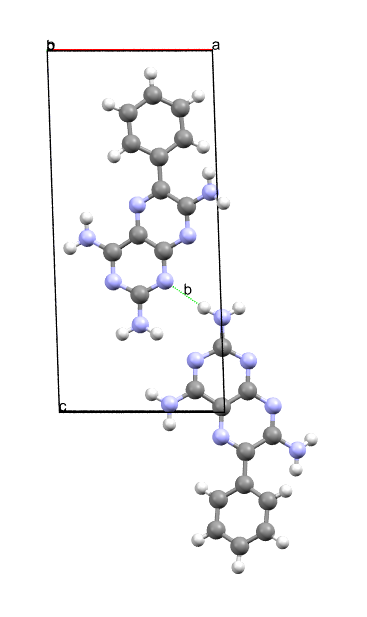 |
| C1,1 (6)•[c] (H4A…N8A) | D1,1(2)•[d] (H6A…N2B) |
| 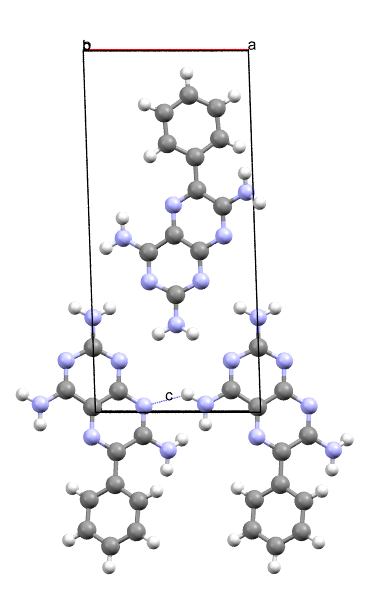 | 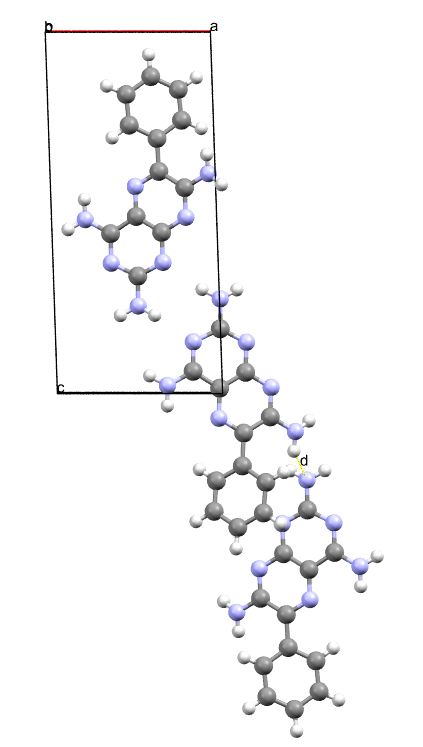 |

**Figure S2**. Unitary motifs of triamterene (1).

| R2,2(8)•[e] (H7A…N8A + H7A…N8A) | D1,1(2)•[f] (H2B…N3A) |
| --- | --- |
| 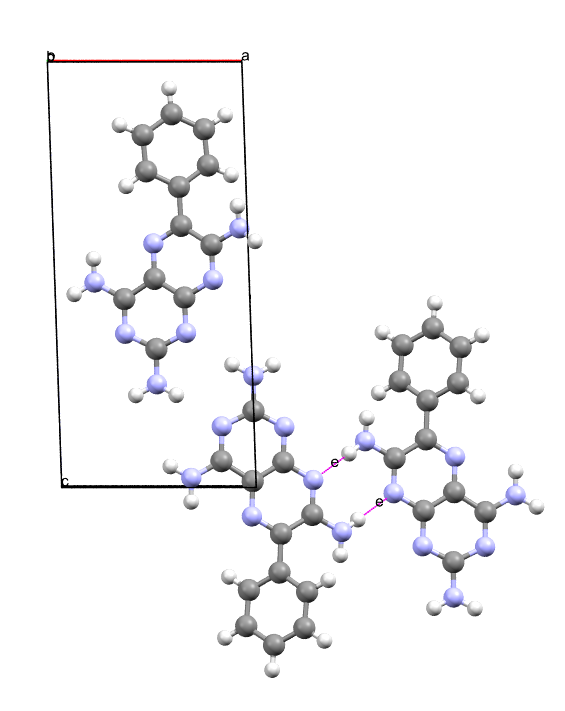 | 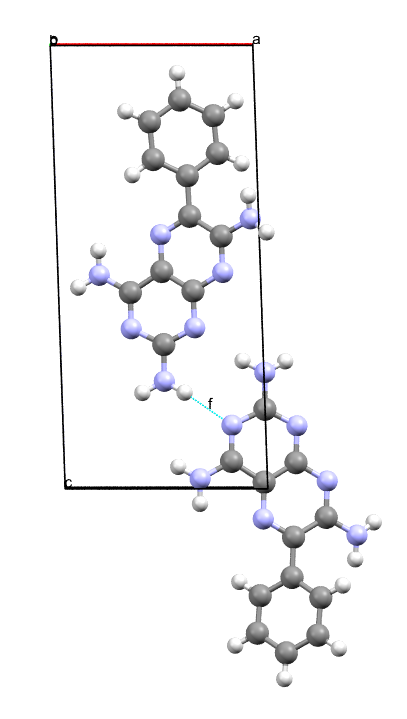 |
| D1,1(2)•[g] (H3B…N1A) | C1,1(6)•[h] (H4B…N8B) |
| 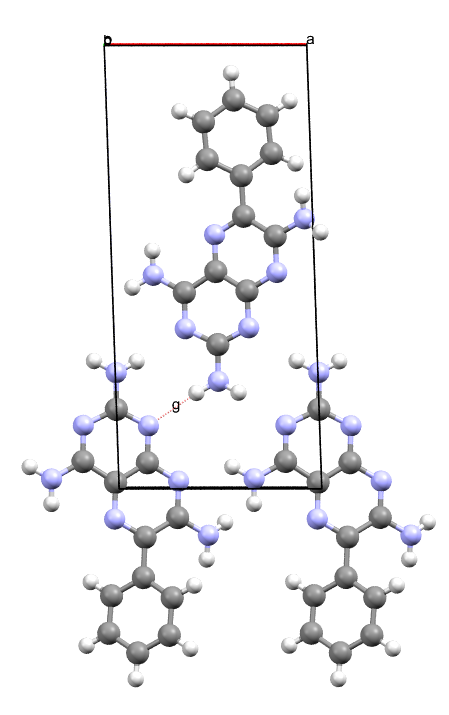 | 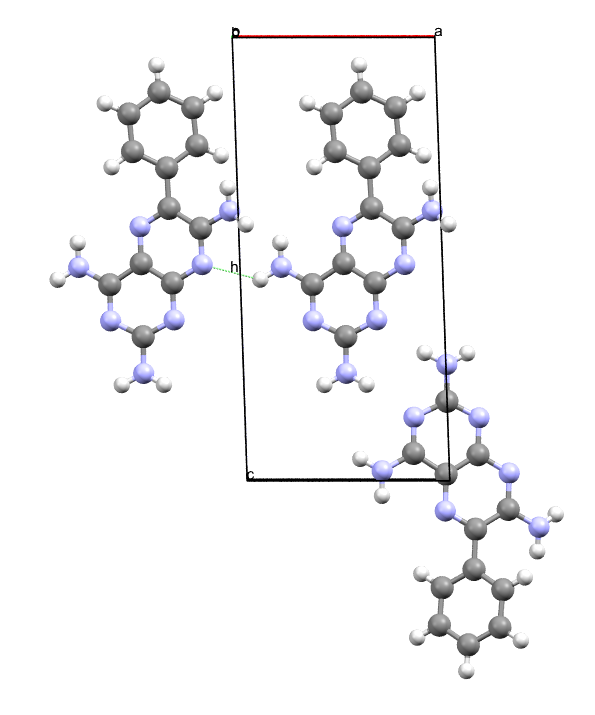 |

**Figure S2 (continued).** Unitary motifs of triamterene (2).

| D1,1(2)•[i] (H6B…N2A) | R2,2(8)•[j] (H7B…N8B + H7B…N8B) |
| --- | --- |
| 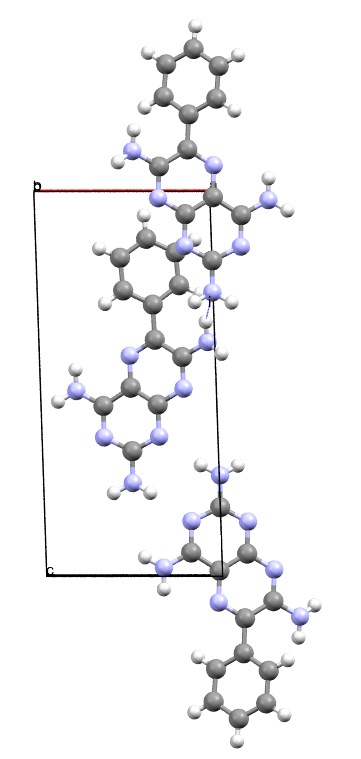 | 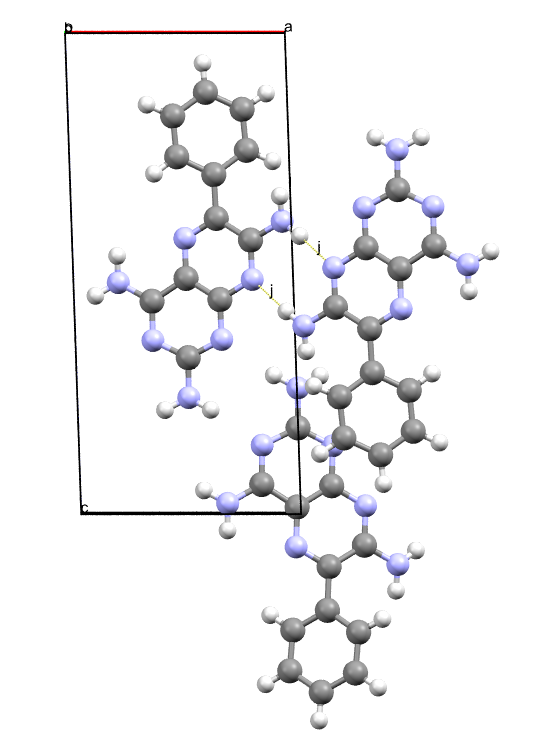 |

**Figure S2 (continued).** Unitary motifs of triamterene (3).

**4. Binary motifs of trimaterene**

| C2,2(6)•[>a<b] | R4,4(24)•[>a<d>a<d] |
| --- | --- |
| 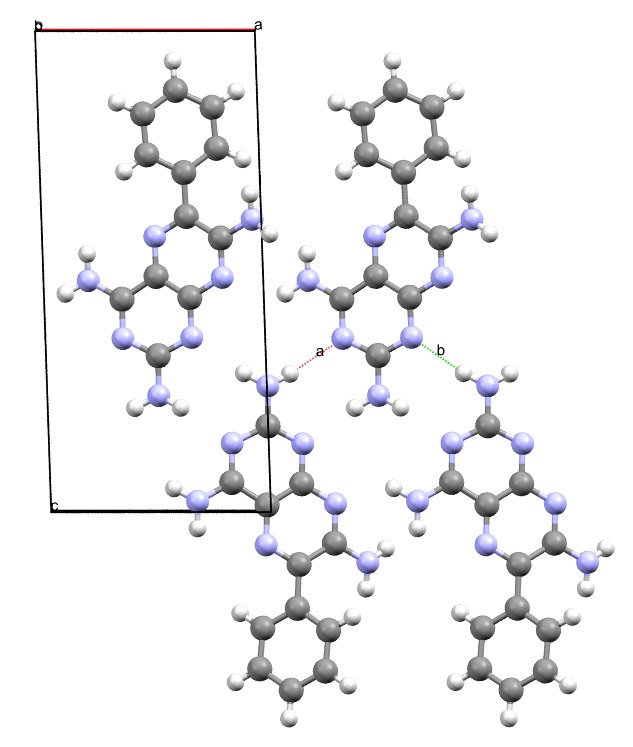 | 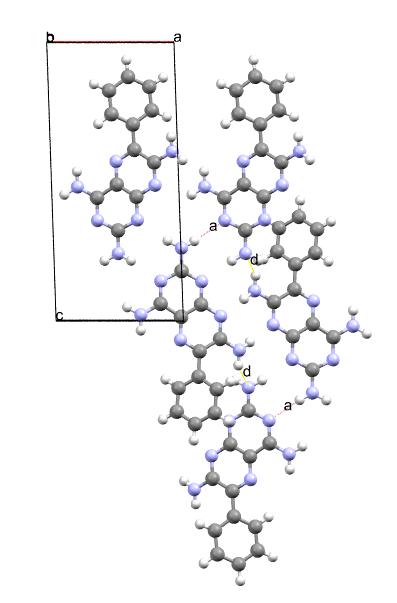 |
| C2,2(8)•[>a>f] | R2,2(8)•[>a>g] |
| 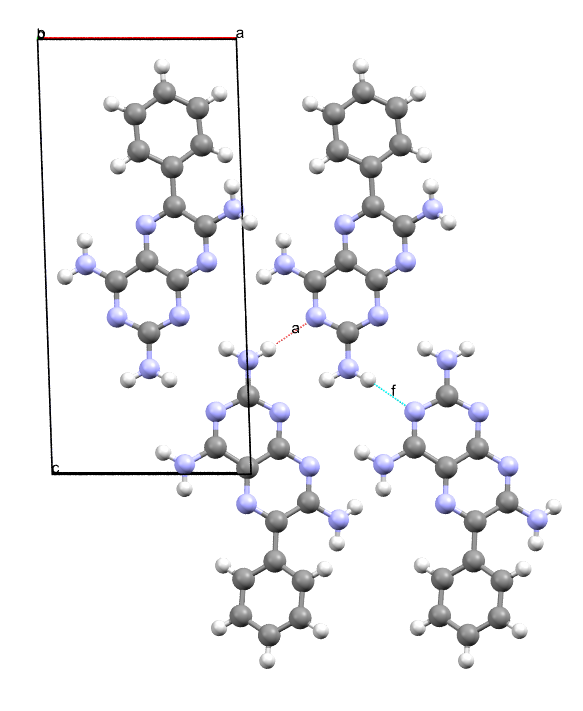 | 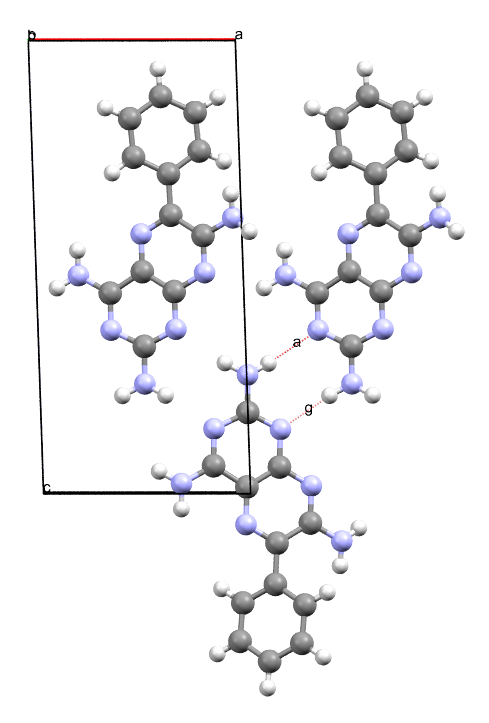 |

**Figure S3.** Binary motifs of triamterene (1).

| R4,4(20)•[>a>i>a>i] | R4,4(24)•[>b<d>b<d] |
| --- | --- |
| 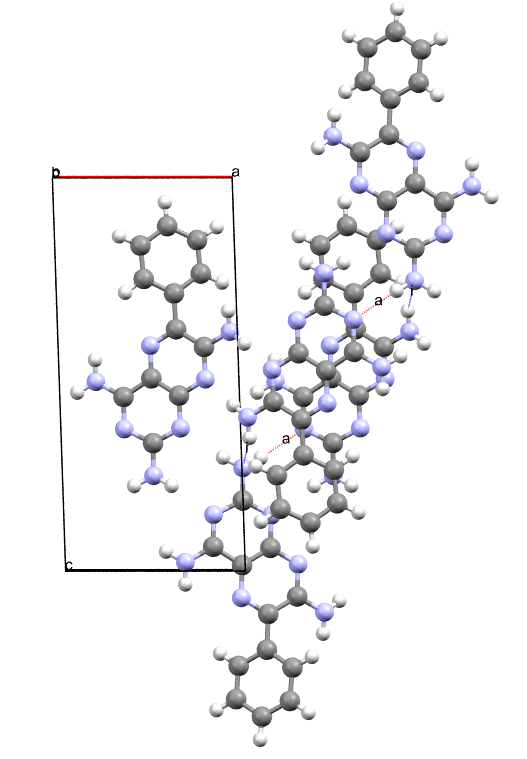 | 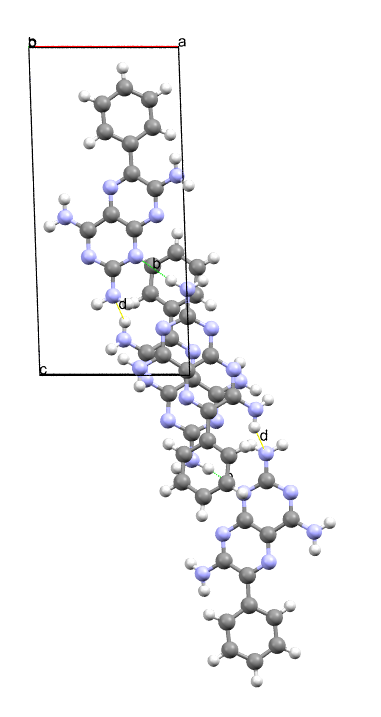 |
| R2,2(8)•[>b>f] | C2,2(8)•[>b>g] |
| 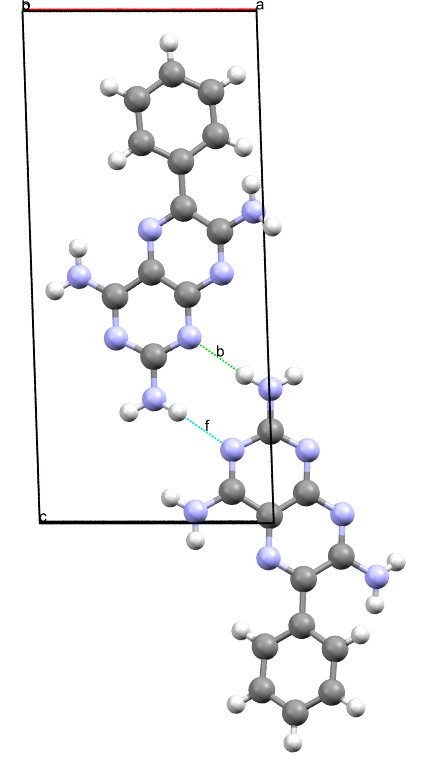 | 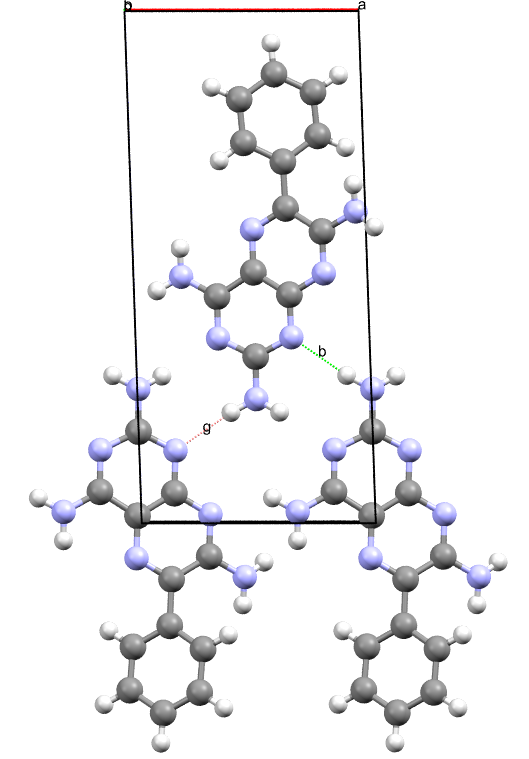 |

**Figure S3 (continued).** Binary motifs of triamterene (2).

| R4,4(16)•[>b>i>b>i] | R2,4(20)•[>c<e>c<e] |
| --- | --- |
| 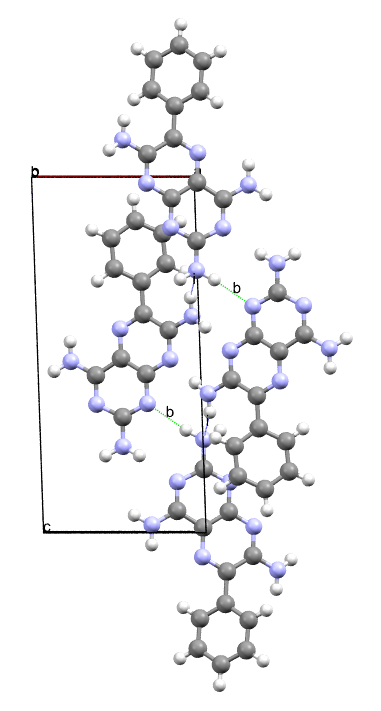 | 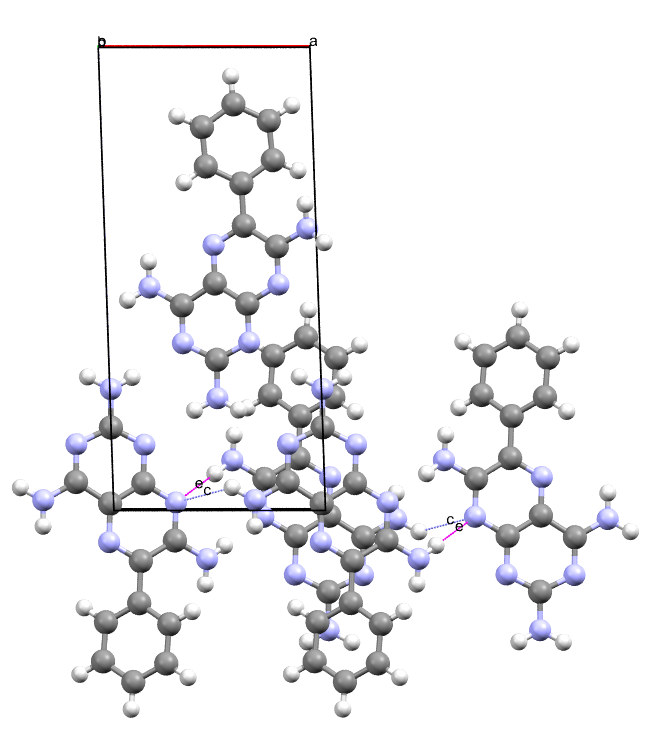 |
| R4,4(20)•[>d>f>d>f] | R4,4(16)•[>d>g>d>g] |
| 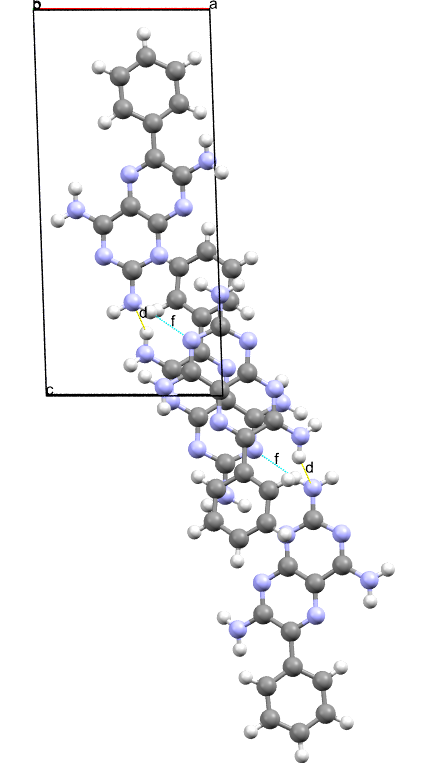 | 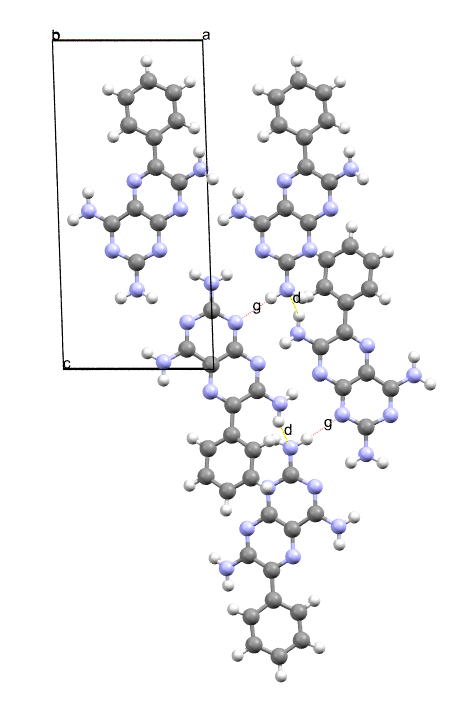 |

**Figure S3 (continued).** Binary motifs of triamterene (3).

| C2,2(16)•[>d>i] | C2,2(6)•[>f<g] |
| --- | --- |
| 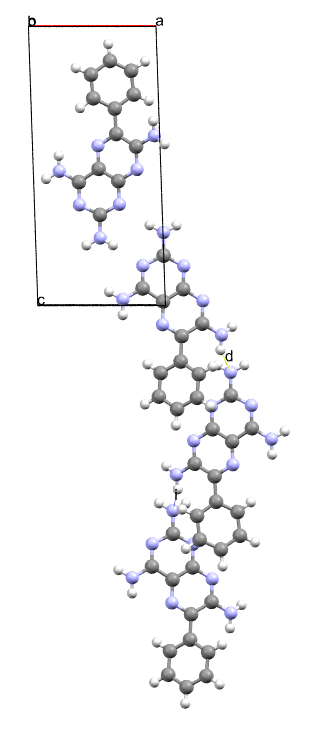 | 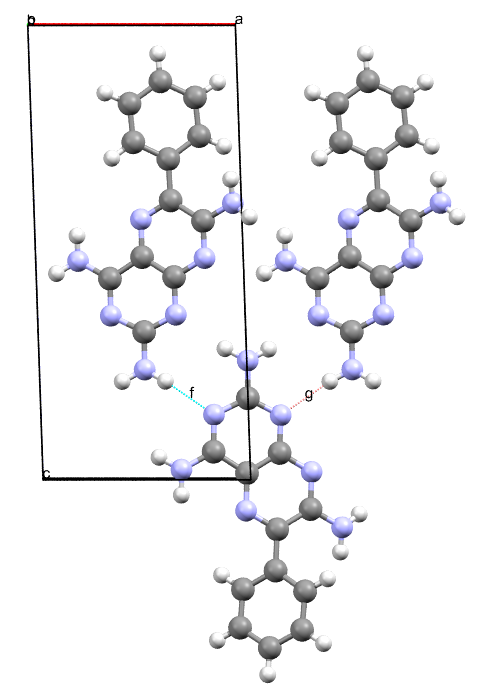 |
| R4,4(24)•[>f<i>f<i] | R4,4(24)•[>g<i>g<i] |
| 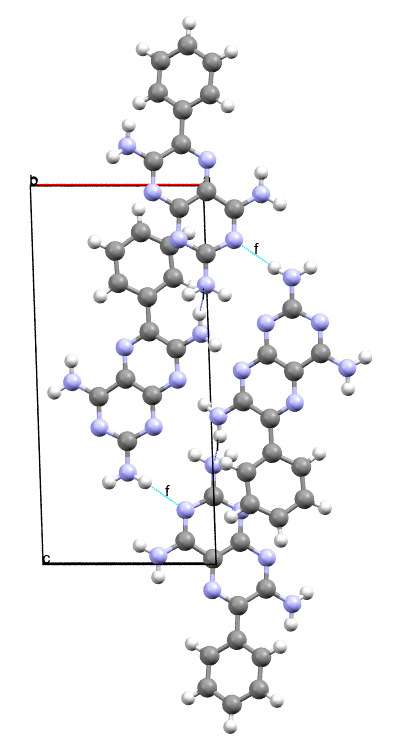 | 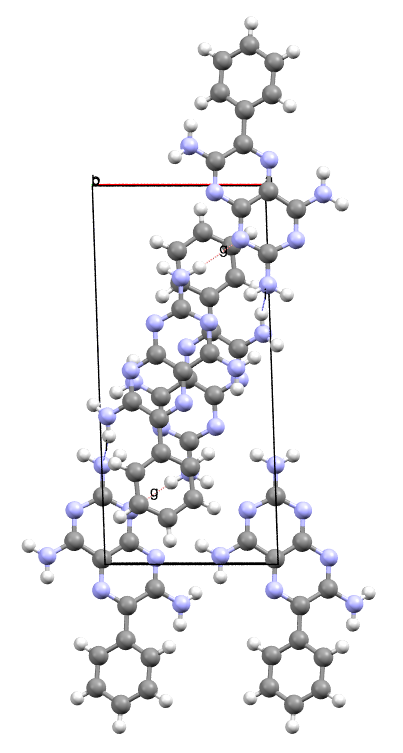 |

**Figure S3 (continued).** Binary motifs of triamterene (4).

| R2,4(20)•[>h<j>h<j] |
| --- |
| 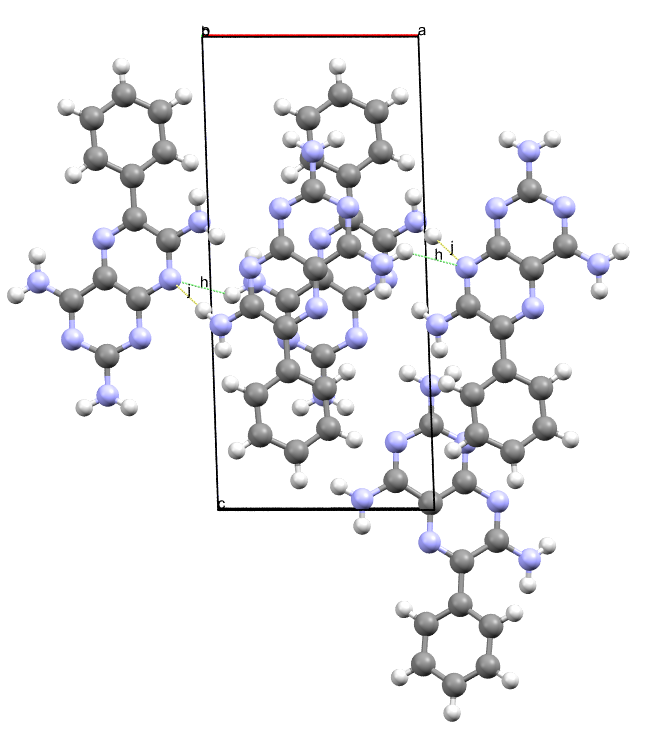 |

**Figure S3 (continued).** Binary motifs of triamterene (5).

-------------------------------------------------------------------
